# The role of China's terrestrial carbon sequestration 2010–2060 in offsetting energy-related CO_2_ emissions

**DOI:** 10.1093/nsr/nwac057

**Published:** 2022-03-25

**Authors:** Yao Huang, Wenjuan Sun, Zhangcai Qin, Wen Zhang, Yongqiang Yu, Tingting Li, Qing Zhang, Guocheng Wang, Lingfei Yu, Yijie Wang, Fan Ding, Ping Zhang

**Affiliations:** State Key Laboratory of Vegetation and Environmental Change, Institute of Botany, Chinese Academy of Sciences, Beijing 100093, China; State Key Laboratory of Atmospheric Boundary Layer Physics and Atmospheric Chemistry, Institute of Atmospheric Physics, Chinese Academy of Sciences, Beijing 100029, China; State Key Laboratory of Vegetation and Environmental Change, Institute of Botany, Chinese Academy of Sciences, Beijing 100093, China; School of Atmospheric Sciences, Sun Yat-sen University, Guangzhou 510275, China; State Key Laboratory of Atmospheric Boundary Layer Physics and Atmospheric Chemistry, Institute of Atmospheric Physics, Chinese Academy of Sciences, Beijing 100029, China; State Key Laboratory of Atmospheric Boundary Layer Physics and Atmospheric Chemistry, Institute of Atmospheric Physics, Chinese Academy of Sciences, Beijing 100029, China; State Key Laboratory of Atmospheric Boundary Layer Physics and Atmospheric Chemistry, Institute of Atmospheric Physics, Chinese Academy of Sciences, Beijing 100029, China; State Key Laboratory of Atmospheric Boundary Layer Physics and Atmospheric Chemistry, Institute of Atmospheric Physics, Chinese Academy of Sciences, Beijing 100029, China; State Key Laboratory of Atmospheric Boundary Layer Physics and Atmospheric Chemistry, Institute of Atmospheric Physics, Chinese Academy of Sciences, Beijing 100029, China; State Key Laboratory of Vegetation and Environmental Change, Institute of Botany, Chinese Academy of Sciences, Beijing 100093, China; State Key Laboratory of Vegetation and Environmental Change, Institute of Botany, Chinese Academy of Sciences, Beijing 100093, China; College of Land and Environment, Shenyang Agricultural University, Shenyang 110866, China; College of New Energy and Environment, Jilin University, Changchun 130021, China

**Keywords:** terrestrial carbon sequestration, natural climate solutions, CO_2_ emission, energy consumption, carbon neutrality

## Abstract

Energy consumption dominates annual CO_2_ emissions in China. It is essential to significantly reduce CO_2_ emissions from energy consumption to reach national carbon neutrality by 2060, while the role of terrestrial carbon sequestration in offsetting energy-related CO_2_ emissions cannot be underestimated. Natural climate solutions (NCS), including improvements in terrestrial carbon sequestration, represent readily deployable options to offset anthropogenic greenhouse gas emissions. However, the extent to which China's terrestrial carbon sequestration in the future, especially when target-oriented managements (TOMs) are implemented, can help to mitigate energy-related CO_2_ emissions is far from certain. By synthesizing available findings and using several parameter-sparse empirical models that have been calibrated and/or fitted against contemporary measurements, we assessed China's terrestrial carbon sequestration over 2010–2060 and its contribution to offsetting national energy-related CO_2_ emissions. We show that terrestrial C sequestration in China will increase from 0.375 ± 0.056 (mean ± standard deviation) Pg C yr^−1^ in the 2010s to 0.458 ± 0.100 Pg C yr^−1^ under RCP2.6 and 0.493 ± 0.108 Pg C yr^−1^ under the RCP4.5 scenario in the 2050s, when TOMs are implemented. The majority of carbon sequestration comes from forest, accounting for 67.8–71.4% of the total amount. China's terrestrial ecosystems can offset 12.2–15.0% and 13.4–17.8% of energy-related peak CO_2_ emissions in 2030 and 2060, respectively. The implementation of TOMs contributes 11.9% of the overall terrestrial carbon sequestration in the 2020s and 23.7% in the 2050s. The most likely strategy to maximize future NCS effectiveness is a full implementation of all applicable cost-effective NCS pathways in China. Our findings highlight the role of terrestrial carbon sequestration in offsetting energy-related CO_2_ emissions and put forward future needs in the context of carbon neutrality.

## Teaser

China's terrestrial ecosystems could sequester carbon at the rates of 0.375 Pg C yr^−1^ in the 2010s and 0.458 ± 0.493 Pg C yr^−1^ in the 2050s. Target-oriented managements contribute 12–24% to the total carbon sequestration. China's terrestrial carbon sequestration in 2060 could offset 13–18% of energy-related peak CO_2_ emissions.

## INTRODUCTION

Atmospheric CO_2_ concentration has increased by 48% since the pre-industrial era [1] and this increase will continue [2]. To achieve the Paris Agreement target of limiting the global temperature rise to well <2°C above the pre-industrial level and to pursue efforts to keep warming at <1.5°C, global efforts are urgently needed to reduce greenhouse gas emissions by 50% in the next 10 years and reach net zero by the 2050s so that the 1.5°C target can be possible [3,4].

Energy consumption dominates CO_2_ emissions in China, accounting for ∼88% of the total annual CO_2_ emission in the year 2010 [5]. Chinese government has pledged to reach peak CO_2_ emissions before 2030 and carbon neutrality before 2060. The CO_2_ emission from energy consumption in 2030 is projected to be 10.2–12.5 Pg CO_2_ yr^−1^, or 2.8–3.4 Pg C yr^−1^ (1 Pg = 10^12^ kg) depending on different scenarios [5]. Coal is still the major energy source in China, contributing 56.8% to the total energy consumption in 2020. Much attention has been paid to CO_2_ mitigation in energy sectors. To reach carbon neutrality, the percentage of coal in energy consumption should be reduced from 43.2−46.0% in 2030 to 9.1−25.2% in 2050 [6], or the percentage of non-fossil energy consumption should be increased from 24.2−28.7% in 2030 to 85% in 2060 [7]. The non-fossil energy includes mainly wind, solar, hydraulic and nuclear. It is a big challenge to reduce fossil energy consumption, especially coal, to reach carbon neutrality in China.

Although it is essential to reduce CO_2_ emissions from energy consumption, the role of terrestrial carbon sequestration cannot be underestimated in carbon neutrality. Natural climate solutions (NCS) or nature-based climate solutions, which promote conservation, restoration and land management improvement to increase terrestrial carbon sequestration or reduce emissions from ecosystems [8], have been well recognized as one of the most effective, readily available mitigation options [9–13]. Global NCS could provide a quarter or more of the cost-effective mitigation needed by 2030 [14]. Reforestation is potentially a large-scale method for sequestrating CO_2_ in the biomass and soils of ecosystems. The carbon (C) sequestration from tropical reforestation between 2020 and 2050 could be increased by 1.55 Pg C at a carbon price of US$20 tCO_2_^−1^ [15]. The NCS could have offset 21% of the net annual emissions of the USA in 2016 [16].

Global terrestrial ecosystems sequestered C and this sequestration has shown an increased trend over the last four decades. The rates of C sequestration by global terrestrial ecosystems were 2.0, 2.6, 2.9 and 3.4 Pg C yr^−1^ during the 1980s, 1990s, 2000s and 2010s, respectively, offsetting 36−41% of fossil CO_2_ emissions [17]. Terrestrial ecosystems in China sequestered C at rates of 0.19–0.26 Pg C yr^−1^ over the 1980s–1990s [18], 0.20–0.33 Pg C yr^−1^ during the 2000s [19–21], offsetting 28–37% and 11–18% of fossil CO_2_ emissions over the 1980s–1990s and the 2000s, respectively. The forest ecosystem plays a dominant role in China's terrestrial C sequestration [19,22]. The implementation of six ecological restoration projects since the late 1970s has significantly increased ecosystem C sequestration in China, contributing 56% to the overall C sequestration across the restored regions [23].

A few studies have estimated the C sequestration from China's terrestrial ecosystems in the future. Under different climate scenarios, model simulations showed terrestrial C sequestration at rates of 0.256–0.397 Pg C yr^−1^ between 2020 and 2060 when the land use remained as in the 1970s [24]. Scientists paid specific attention to future C sequestration in forests. Based on age-dependent changes in the forest biomass, the vegetation C sequestration was estimated to be 0.145 Pg C yr^−1^ between 2000 and 2050 [25]; 0.176 Pg C yr^−1^ between 2008 and 2050, taking afforestation into consideration [26]; and 0.28 −0.42 Pg C yr^−1^ between 2010 and 2050, with forest area kept unchanged [27]. However, these age-dependent estimates [25–27] neither included below-ground biomass nor took CO_2_ fertilization into account. More recent studies have suggested that the historic land C sinks were underestimated in current models due to an undervalued CO_2_ fertilization effect [28]. Multi-model estimates suggested that an increase in atmospheric CO_2_ of 100 ppm would sequester 3.1–3.5 Pg C yr^−1^ in global terrestrial ecosystems [29,30]. Enhanced global terrestrial GPP (gross primary productivity) due to CO_2_ fertilization reached 1.8 Pg C yr^−1^ during 2001–2014 [31]. Nevertheless, the CO_2_ fertilization effect on C sequestration can be reduced in a mature forest [32] and in plants growing in nutrient-poor soils [33,34], and can even be diminished over time when plants grow under long-term CO_2_ enrichment conditions [35,36].

Management practices in China have shown that forest plantations increased C sequestration [18,23,37], grazing exclusion in degraded grasslands promoted C storage [38,39] and the enhancement of residue retention in croplands improved soil C sequestration [40,41]. China has proposed a series of target-oriented plans associated with ecological conservation and restoration [42–45], which will undoubtedly promote future terrestrial C sequestration nationwide.

C sequestration in China's terrestrial ecosystems over the past decades has been well recognized and quantified [18,19]. Under different climate change scenarios, some model simulations projected future C sequestration in forests [46], cropland soils [47] and grasslands [48] in China. However, the extent to which China's terrestrial C sequestration in the future, especially when target-oriented managements (TOMs) are implemented, can help to mitigate energy-related CO_2_ emissions is far from certain due to a lack of integrated investigations. This limits our overall evaluation of future C sequestration in China's terrestrial ecosystems and the role in offsetting energy-related CO_2_ emissions. Here, we focus on China's terrestrial ecosystems, including forests, shrubland, grassland, cropland and wetland. By synthesizing available findings and using several parameter-sparse empirical models, we first estimate the C sequestration in China's terrestrial ecosystems over the period 2010–2060 by taking into consideration CO_2_ fertilization impacts, TOMs and below-ground biomass. We then evaluate the contribution of terrestrial C sequestration to offsetting energy-related CO_2_ emissions in China over the period 2010–2060. We expect that our estimates of future terrestrial C sequestration in China could serve as the basis for national policy making and further research in this area.

## RESULTS

### Carbon sequestration during the period 2010–2060

By synthesizing available findings and using several parameter-sparse empirical models [Equations (1)–(8) and Equations (S1)–(S9) in Supplementary Methods] that have been calibrated and/or fitted against contemporary measurements, we estimated China's terrestrial C sequestration in 2010–2060.

**Figure 1.**
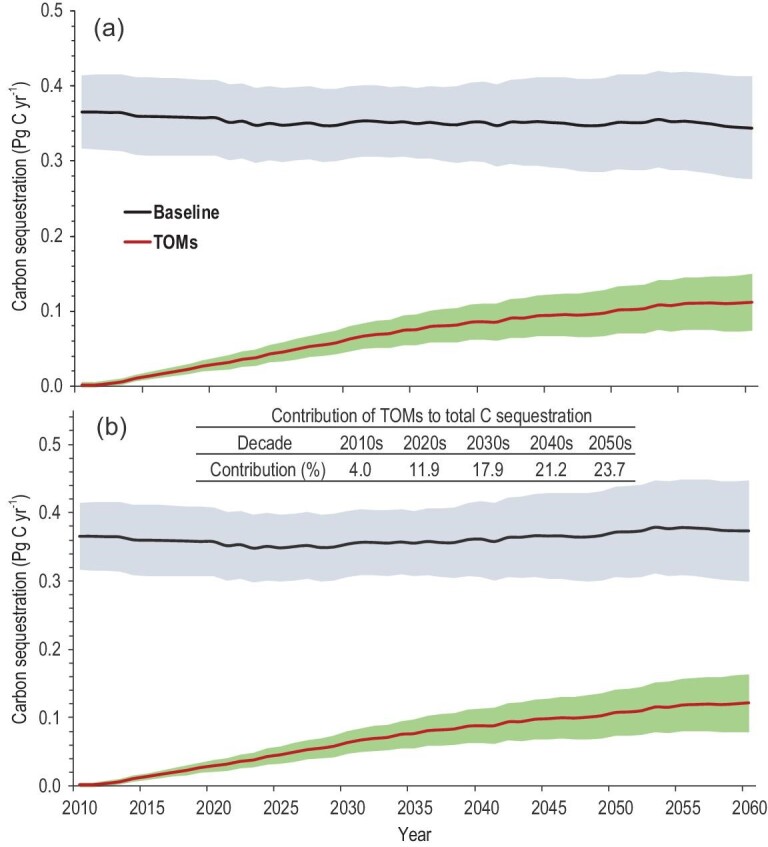
Estimated C sequestration in terrestrial ecosystems under RCP2.6 (a) and RCP4.5 (b). Shaded area shows the aggregated standard deviation.

Terrestrial C sequestration in China at baseline shows a slight decrease under RCP2.6 (Fig. 1a) but remains relatively stable under RCP4.5 (Fig. 1b) from 2010 to 2060. The TOMs promote C sequestration with time (Fig. 1). An overall increase in China's terrestrial C sequestration was estimated to be from 0.375 ± 0.056 [mean ± standard deviation (SD)] Pg C yr^−1^ in the 2010s to 0.458 ± 0.100 Pg C yr^−1^ under RCP2.6 and 0.493 ± 0.108 Pg C yr^−1^ under an RCP4.5 scenario in the 2050s (Table 1). In 2030 and 2060, terrestrial ecosystems can sequester 0.415 ± 0.064 and 0.456 ± 0.105 Pg C yr^–1^ under RCP2.6, and 0.417 ± 0.065 and 0.496 ± 0.114 Pg C yr^–1^ under an RCP4.5 scenario. Terrestrial C sequestration under RCP4.5 is 7.6% higher than that under an RCP2.6 scenario in the 2050s (Table 1). The contribution of TOMs to the total C sequestration accounts for 4.0% in the 2010s and 23.7% in the 2050s (Fig. 1b and Table S1), indicating the importance of TOMs in C sequestration. The vegetation C sequestration, on average, contributes 52.5–54.5% of the total C sequestration (Table 1).

**Table 1.** Estimated C sequestration rate (Pg C yr^−1^) under two climate scenarios.

		RCP2.6		RCP4.5	
Component	Decade	Mean	SD^a^	Mean	SD
Vegetation	2010s	0.203	0.027	0.203	0.027
	2020s	0.210	0.029	0.210	0.029
	2030s	0.225	0.032	0.229	0.032
	2040s	0.237	0.050	0.247	0.052
	2050s	0.250	0.061	0.267	0.065
Soil	2010s	0.173	0.029	0.173	0.029
	2020s	0.187	0.033	0.187	0.033
	2030s	0.203	0.035	0.207	0.036
	2040s	0.207	0.038	0.217	0.040
	2050s	0.209	0.039	0.226	0.043
Total	2010s	0.375	0.056	0.375	0.056
	2020s	0.397	0.062	0.397	0.062
	2030s	0.428	0.067	0.435	0.068
	2040s	0.444	0.088	0.464	0.092
	2050s	0.458	0.100	0.493	0.108

^a^The mean value of the aggregated standard deviation (SD) for a certain decade.

Forests can sequester 0.260–0.350 Pg C yr^−1^ between the 2010s and 2050s. Vegetation C sequestration accounts for 72.4% of the total C sequestration. The TOM (area increase via afforestation) contributes 4.6% in the 2010s and 25.2% in the 2050s to the total C sequestration (Table S2). During the period of 2010s–2050s, shrubland can sequester 0.027–0.028 Pg C yr^−1^ at baseline, but the C sequestration will decrease (Table S3) due to part of the shrubland area being transformed to forest (please see Methods). Carbon sequestration in grassland is estimated to be 0.018–0.031 Pg C yr^−1^. The contribution of TOM (restoration via exclosure from grazing) to the total C sequestration will increase from 13.9% in the 2020s to 30.5% in the 2050s (Table S4).

Cropland soils can sequester C at rates of 0.043–0.059 Pg C yr^−1^. The increase in crop residue retention and the expansion of no-till areas (Methods) contribute 10.8% in the 2010s and 48.7% in the 2050s to the total C sequestration (Table S5). Carbon sequestration in wetland soils is estimated to be 0.031–0.043 Pg C yr^−1^ between the 2010s and 2050s (Table S6), 2.7–21.7% of which is attributed to an increase in area through restoration (Methods).

The majority of C sequestration comes from forests, accounting for 67.8–71.4% of the total amount. Cropland soils, wetland soils, grassland and shrubland contribute 11.5–13.3%, 8.1–8.8%, 4.8–6.0% and 2.4–6.5%, respectively, to the total C sequestration when the TOMs are deployed (Table 2).

**Table 2.** Contribution of different ecosystems to C sequestration (%).

Item	Decade	Forest	Shrubland	Grassland	Cropland	Wetland
Baseline	2010s	68.7	7.4	5.0	10.6	8.3
	2020s	68.3	7.9	5.3	9.8	8.7
	2030s	69.1	8.0	5.4	8.7	8.9
	2040s	69.4	8.0	5.5	8.1	9.0
	2050s	69.9	8.0	5.5	7.7	9.0
Baseline + TOMs	2010s	69.2	6.5	4.8	11.5	8.1
	2020s	67.8	5.4	5.4	12.8	8.6
	2030s	67.9	4.2	5.9	13.3	8.7
	2040s	69.4	3.3	6.0	12.5	8.7
	2050s	71.4	2.4	6.0	11.5	8.8

### Contribution of C sequestration to offsetting energy-related CO_2_ emissions

According to the People's Republic of China Third National Communication on Climate Change [5], the energy-related CO_2_ emissions in China were 7.6 Pg CO_2_ yr^−1^, or 2.1 Pg C yr^−1^, in 2010 and are projected to be 10.2–12.5 Pg CO_2_ yr^−1^, or 2.8–3.4 Pg C yr^−1^, in 2030 (peak emissions), depending on different policy scenarios (see details in Table S7). Using Equation (10), we calculated the contribution of terrestrial C sequestration to offsetting energy-related CO_2_ emissions.

**Figure 2.**
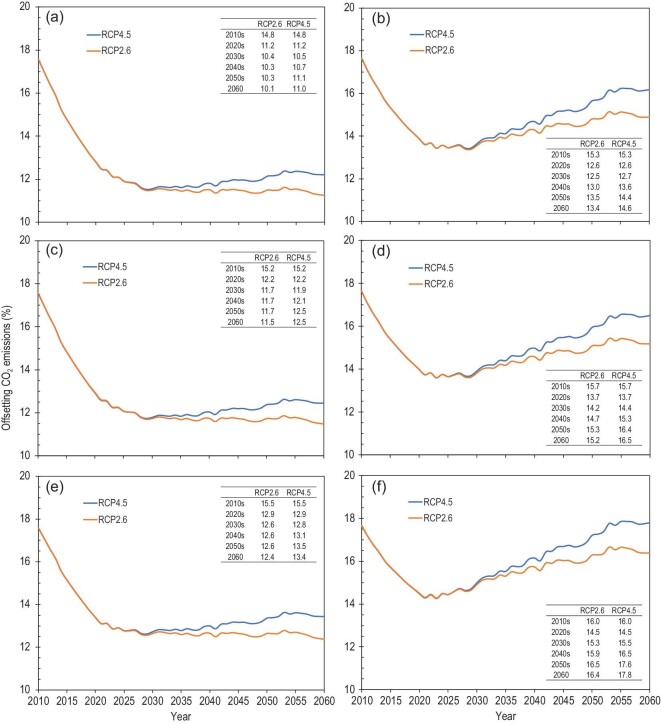
Percentage of offsetting energy-related CO_2_ emissions via (a, c, e) terrestrial C sequestration at baseline and (b, d, f) baseline + target-oriented managements. The scenarios of energy-related CO_2_ emissions are (a, b) reference, (c, d) policy-I and (e, f) policy-II. Supplementary Methods and Table S7 show detailed information about energy-related CO_2_ emission under different scenarios.

Terrestrial C sequestration at baseline can offset 14.8–15.5% of energy-related CO_2_ emissions in the 2010s, but this offsetting effect will decline to 10.3–13.5% of energy-related peak CO_2_ emissions in the 2050s depending on CO_2_ emission scenarios (Fig. 2a, c and e). This is due not only to an increase in CO_2_ emissions from 2010 to 2030 (Table S7) but also to a lack of TOMs in terrestrial ecosystems. In contrast, TOMs can largely promote the offsetting effect. Terrestrial C sequestration can offset 15.3–16.0% of energy-related CO_2_ emissions in the 2010s and 13.5–17.6% of energy-related peak CO_2_ emissions in the 2050s when the TOMs are put into practice (Fig. 2b, d and f).

The total C sequestration can offset 12.2–15.0%, 12.9–16.2% and 13.3–17.2% of energy-related peak CO_2_ emissions in 2030, 2040 and 2050 under different scenarios, respectively. In 2060, terrestrial C sequestration can offset 13.4–16.4% and 14.6–17.8% of energy-related peak CO_2_ emissions under RCP2.6 and RCP4.5 scenarios (Fig. 2b, d and f), while the offsetting at baseline (Fig. 2a, c and e) is ∼24% lower than those with TOMs.

## DISCUSSION

### Achievability and implications

Forest-based NCS have experienced growing interest in recent years as a potentially major contributor to meeting Paris Agreement carbon targets [13]. Table 2 shows that the forest sector accounts for the vast majority of terrestrial C sequestration. The TOM contributes 25.2% to the forest C sequestration in the 2050s (Table S2). Forest areas increased from 195.89 Mha (million hectares) in 2008 to 220.57 Mha in 2020 at a mean rate of 2.03 Mha yr^−1^ (Fig. S1a). Meanwhile, forest coverage increased at a mean rate of 0.23% yr^−1^ (Fig. S1b). As a target, the forest area is proposed to increase by 47 Mha on the basis of 2020 and the forest coverage will reach and stabilize at >26% by 2050 (Fig. S1b). Accordingly, the increase rates would be 1.57 Mha yr^−1^ for the area and 0.10% yr^−1^ for the coverage. From practice over the past 10 years, the increase in forest areas and thus the promotion of C sequestration is achievable in the future.

There is a general agreement that the technologically achievable soil organic carbon (SOC) sequestration via management practices is significant in global croplands [49,50]. Cropland soils can contribute 11.5−13.3% to the total C sequestration in China (Table 2), which is substantially attributed to TOM (Table S5). The current SOC density (0–30 cm) in China's cropland is ∼37.4 Mg C ha^−1^ [51], lower than the global mean of ∼53 Mg C ha^−1^ [52]. The SOC density is expected to reach 57.6−58.5 Mg C ha^−1^ in 2060 if TOM is practiced. Taking the global mean as a reference, the achievability of SOC sequestration is most likely possible in the future.

We assumed that a part of cropland will be transformed to wetland between 2010 and 2030. The cropland area will be reduced from 130 Mha in 2010 to 128.6 Mha in 2030 (Table S8), corresponding to a 1.1% reduction, or 0.05% per year. In light of the National DATA created by the National Bureau of Statistics of China (https://data.stats.gov.cn/), the production of grain yield in China, on average, increased by 1.9% per year over the period 2011–2020. The 0.05% yr^−1^ reduction in cropland area may not affect food security.

Grazing exclusion (GE) is well recognized to be an important strategy for restoring degraded grasslands and promoting C storage [38]. The implementation of the Grassland Ecological Compensation Policy in China over 2008−2013 leads to a 3.2% increase in NDVI (normalized difference vegetation index) [53]. Analysis of the data from the China Environmental Status Bulletin shows that the production of hay from 2005 to 2017 increased at a rate of 2.97 Tg DW yr^−1^ (1 Tg = 10^9^ kg; DW represents dry weight) in China's grassland (Fig. S2a), which is substantially attributed to GE (Fig. S2b). Improvements in hay production can lead to a synchronous increase in below-ground biomass and thus SOC. The TOM can contribute 30.5% to the grassland C sequestration in the 2050s (Table S4). The accumulative area of GE over 2003−2012 was 43.54 Mha. GE in an additional 57.5 Mha of the moderately and heavily degraded area between 2021 and 2060 (Table 3) is very likely to improve grassland C sequestration.

**Table 3.** Scenarios for target-oriented management in different ecosystems.

Ecosystem	Baseline	Target-oriented management
Forest	The observed area from 2011 to 2020 was adopted, and the area remains unchanged after 2020.	The area will increase by 47 million hectors in 2050 based on the 2020 value [45], 60% and 40% of which come from shrubland and grassland, respectively.
Shrubland	The area remains unchanged.	Part of the area will be transformed to forest after 2020.
Grassland	The area remains unchanged.	Part of the area will be transformed to forest after 2020. 50% of the moderately and above degraded area (57.5 Mha) in Tibet, Inner Mongolia, Xinjiang, Qinghai and Gansu will be restored by enclosure between 2021 and 2060.
Cropland	The area remains unchanged. Crop yield, the proportion of crop residue retention, manure input and no-till practice remain at the 2010s level.	Part of the area will be transformed to wetland after 2010. Crop yield will increase by 0.5% per year between 2021 and 2060 based on the mean of 2016−2020 values. The proportion of crop residue retention will increase from the baseline to 60% in 2060 and manure input will remain unchanged. The proportion of no-till area will increase from the baseline to 30% in 2060.
Wetland	The area remains unchanged.	The area increased by 474.5 × 10^3^ ha between 2010 and 2020, and will increase by 929.5 × 10^3 ^ha between 2021 and 2030 [111] based on the 2020 value via wetland restoration.

China emitted 28% of global fossil CO_2_ emissions in 2019 [17] and the fossil CO_2_ emissions are likely to keep increasing before reaching a peak in 2030 (Table S7). The NCS should be taken into consideration to meet the 2060 carbon neutrality goal [54], although much attention has been paid to reducing the fossil CO_2_ emissions [6,7,55]. China's terrestrial ecosystems in 2060 can sequester 0.456 and 0.496 Pg C yr^–1^ under RCP2.6 and RCP4.5 scenarios, respectively, suggesting that there would be room for 0.456–0.496 Pg C yr^–1^ emissions in energy sectors in 2060, corresponding to 13.4–17.8% of energy-related peak CO_2_ emissions. There is no doubt that terrestrial C sequestration in China will greatly reduce the pressure on cutting CO_2_ emissions from energy consumption. We have reason to believe that the NCS in the next 40 years can and should play an irreplaceable role in accomplishing the carbon neutrality goal by 2060.

### Uncertainties and limitations

Unlike previous model simulation studies on global [30,56,57] or ecosystem-specific C sequestration [46,58], we synthesized and analysed available findings and used several parameter-sparse empirical models (see Methods and Supplementary Methods) to make our estimates of C sequestration, which makes it difficult to calculate uncertainties. However, the large SD in the estimates of C sequestration (Table 1) suggests higher uncertainties of the estimates. The uncertainties may principally come from several sources, including the usage of various estimates from different researchers, simplifications due to a lack of information, the assumptions of TOMs and imperfect empirical models.

Very few investigations have focused on future forest C sequestration in China. Existing estimates of age-dependent C sequestration in forest above-ground biomass are divergent from different researchers, with a range from 500 [25] to 649 kg C ha^−1^ yr^−1^ [26] over the period 2010–2050 without CO_2_ fertilization. It is widely accepted that the accumulation of forest biomass tends to slow down with stand age and thus the rates of C sequestration decrease accordingly [22,25,27]. The available findings [25,26,59] used in this study have also taken into consideration forest ages. Without taking the CO_2_ fertilization effect into consideration, the forest biomass C sequestration in the 2050s will be 8.7 ± 5.7% lower than in the 2010s at baseline. This reduction in C sequestration with time is attributed to increasing forest age, although large variations exist in the reduction between individual studies. These divergent estimates [25,26,59] used in this study would inevitably introduce uncertainties into our estimates of forest C sequestration. We estimated C sequestration in below-ground biomass using a mean value of the root:shoot ratio in forests [22]. This might have also induced uncertainties, since the root:shoot ratio depends not only on forest types, but also on climates [60].

We assumed a linear increase in forest area, the area of GE and the proportion of crop residue retention over the period 2021–2060 under TOMs (see Supplementary Methods) due to a lack of information. This increase may not occur linearly but may vary from year to year, depending on local implementation of TOMs. The linear simplification would lead to uncertainties in our annual estimates.

Evidence suggests a substantial increase in global photosynthesis since pre-industrial times. Elevated CO_2_ is likely responsible for about half of the increase [61,62]. Based on available studies on the response of GPP [31,63] and NPP (net primary production) [64] to elevated CO_2_, we used a value of a 0.13% increase in C sequestration per rising ppm of CO_2_ to quantify the CO_2_ fertilization effect [Equation (4)]. An ensemble of models showed that the relationship between the amplitude of the CO_2_ seasonal cycle and the magnitude of CO_2_ fertilization of GPP is almost linear across the entire ensemble of models [63]. The historical CO_2_ fertilization effect on global GPP was also linearly increased with the atmospheric CO_2_ concentration over the period 1901–2010 [65]. Changes in the residual terrestrial C sink from the global budget showed a linear trend over the period of the 1960s to 2000s [29]. These findings [29,63,65] support our linear quantification of the CO_2_ fertilization effect. Moreover, data from multi-model estimates of future biomass C sequestration in China's forests [46] showed a linear correlation between the biomass C change and the atmospheric CO_2_, with a CO_2_ fertilization effect of 0.21% ppm^–1^ under the RCP2.6, RCP4.5 and RCP8.5 scenarios, which is higher than the 0.13% ppm^–1^ used in this study [Equation (4)].

The CO_2_ fertilization effect is now widely acknowledged [29–31,65,66], whereas nutrients may constrain the response of terrestrial C sequestration to elevated CO_2_ [32–34]. Our quantification of the CO_2_ fertilization effect [Equation (4)] did not take nutrients limitation into consideration, which might have yielded a bias in the estimates of terrestrial C sequestration. Without taking the CO_2_ fertilization effect into consideration, China's terrestrial ecosystems could sequester 0.427 Pg C yr^–1^ in 2060, corresponding to 12.6–15.3% of energy-related peak CO_2_ emissions.

To quantify the impacts of TOMs on C sequestration, several parameter-sparse empirical models were established based on available observations (see Supplementary Methods). Insufficient data used to establish empirical models led to an incomplete understanding of critical processes, though these empirical models [Equations (S1), (S4), (S6) and (S8) in Supplementary Methods] are statistically significant. Parameter-sparse empirical models fitted against measurements can be accurate for the contemporary period, whereas they may not capture additional processes that become important at higher levels of CO_2_ and climate change, especially extreme events. It is necessary to make filed observations under TOMs across a wider domain with various climates, soils and practices to better understand the impacts of TOMs on C sequestration, and thus reduce the uncertainties in the estimates of C sequestration.

We estimated terrestrial C sequestration at the national scale, but its spatial distribution is unclear due to a lack of detailed information. For instance, we simply do not know where the forest area will increase from 2020 to 2060, though the national target is certain [45]. Similar to forests, the TOM via GE is proposed for grassland, but we are unable to identify the location and size in the future. We are aware that the effects of TOMs implementation on C sequestration are variable, depending on climates and soils [23,38]. To objectively estimate spatially specific C sequestration, particularly under the TOMs, a detailed spatio-temporal distribution of land-cover change and the area of TOMs should be clearly projected.

Climate change accelerates both the magnitude and frequency of extreme events such as flooding, drought and storms [67], leading to reduction in terrestrial C sequestration [68–70]. Ignoring the impact of future extreme events on C sequestration is also a limitation of this study. To obtain reliable estimates of future C sequestration, a better understanding and descriptions of both the occurrence of climate extremes and the ecosystem carbon-cycle processes that are triggered by climate extremes need to be achieved [68].

### Future needs

Changes in climate will continue with increasing atmospheric CO_2_ and other greenhouse gases. The effect of increasing atmospheric CO_2_ concentrations on plant growth can be directly measured only at site-specific experiments such as FACE (free-air CO_2_ enrichment) experiments [33,71–73]. To quantify the impacts at the plant and ecosystem levels, researchers rely on other estimates taken from models or FACE experiments, or use proxy data from satellite images [74]. However, differing projections of the C amount absorbed by plants in the future have emerged from the terrestrial biosphere models [74]. Although the effect of CO_2_ on global ecosystem productivity is well recognized, the estimated size of the effect spans an order of magnitude across studies [62], which greatly impedes the ability of models to project future C sequestration [75].

Combining multiple models with data [66] and using model ensembles [29,30] are expected to improve accuracy of C sequestration estimates and thus reduce uncertainties. Although empirical models [Equations (S1), (S4), (S6) and (S8) in Supplementary Methods] can reasonably capture C changes in the contemporary period to support the estimation of terrestrial C sequestration, attention should be paid to several aspects in relation to model projections of future C sequestration in China's terrestrial ecosystems. First, China has proposed a series of target-oriented plans associated with ecological conservation and restoration [42–45]. The region-specific managements associated with these plans should be incorporated into the models to better capture the changes in terrestrial C sequestration. Second, Earth system models (ESMs) simulate physical, chemical and biological processes that underlie climate [76], which are likely to have strong predictive capability of terrestrial C sequestration when extrapolating to climates and human disturbances that have not yet occurred. However, ESMs cannot yet represent the rich ecological detail needed to capture spatial heterogeneity at local scales [76]. Further research is needed to better translate observations into abstract model representations [76] and take the impacts of extreme climate events [68,77] and nutrients limitation [33,34,78] into consideration in models with high spatial resolution. Third, it is critical that the ensemble models such as DGVMs [79] should be widely validated and calibrated with independent observations from country-specific or region-specific field locations that are representative of climate, soil and managements [80], especially in China's terrestrial ecosystems. The fluctuation among model outputs [81–83] should also be assessed to further improve the model performance. It is expected that the improvement of process-level models can capture important features of changing environments and managements with high spatial resolution, lending support to projecting terrestrial C sequestration in China.

Biodiversity experiments have shown that plant diversity promotes plant productivity [84–86] and SOC [87] through niche partitioning among species. Forest above-ground biomass C accumulation in 16-species mixtures was over twice the amount of C observed in average monocultures after 8 years [88]. In subtropical China, the log-transformed forest NPP is positively correlated with species richness [89]. Above-ground woody biomass C and SOC storage in mixed-species broadleaved forests were 82–100% and 11–38% higher than those in single-species broadleaved forests, respectively [90]. Total biomass in the planted forests with a coniferous–broadleaf mixture was 72–77% higher than in those with single species 34 years after the establishment of the plantations [91]. Nonetheless, the improvement in forest biomass C storage in plantations with mixed species depends also on the selection of species. For example, total biomass storage in the mixed *Citrus hystrix* and *Pinus massoniana* plantations was 67% higher than in monoculture *C. hystrix* but 27% lower than in monoculture *P. massoniana* plantations [92]. China is one of the five most forest-rich countries [93]. The increase in forest area is generally achieved through afforestation. Carbon sequestration in planted forest is expected to be promoted when multi-species mixtures or mono species with high productivity are planted in China's afforestation. Moreover, attention should be also paid to region-specific investigations by taking into consideration soils and climates. Appropriate plantation practice will not only be conducive to improving Chinese forest C sequestration, but also likely to provide lessons to other countries worldwide.

Grazing intensity significantly affects below-ground C and nitrogen (N) cycling in grasslands. Heavy grazing decreases soil C and N pool sizes [94]. In a desert steppe in Inner Mongolia, below-ground biomass (BGB) in light grazing (LG) plots was significantly higher than in moderate (MG) and heavy grazing (HG) plots but no significant difference was observed between MG and HG plots over a 10-year experiment [95]. However, the BGB did not show significant difference between LG and MG plots under the same grazing intensity treatments in a desert steppe, though the BGB in HG plots was significantly lower than in LG and MG plots over a 12-year experiment [96]. The carrying capacity of China's grasslands is region-specific and widely variable. In Inner Mongolia, for instance, the carrying capacity was suggested to be 1.0–2.2 sheep units (SU) ha^–1^ for the western desert steppe and 1.8–4.0 SU ha^–1^ for the eastern higher-rainfall meadow steppe [97], suggesting that carrying capacity is related to grassland types, climates and soils. An appropriate stocking density fit to carrying capacity is expected to promote grassland C sequestration [12]. Although we are aware of the possible influence of reduced grazing intensity on C sequestration, an accurate estimate will be hard without knowing detailed information on the carrying capacity and corresponding overgrazing area. To better understand the role of grazing intensity in C sequestration nationwide, future research should focus on identifying historical and spatial changes in carrying capacity and overgrazing, and on determining the rates of C sequestration across different grassland types.

Urban greenery not only improves air quality but also promotes C sequestration [98]. The urban forests in the USA sequestered C at a rate of 0.037 Pg C yr^−1^ with a total of 5.5 billion trees [99]. The C sequestration of street trees in Beijing's urban districts was 3.1 Gg C yr^−1^ (1 Gg = 10^6^ kg) in 2014 [100]. The green space in China's urban area increased from 2.13 Mha in 2010 to 3.15 Mha in 2019 (https://data.stats.gov.cn/) and the urbanization will continue in the future. The increasing green space in urban areas should contribute to terrestrial C sequestration but an accurate estimate is still lacking. It is thus urgently required to assess and project the C sequestration in China's urban trees.

There is evidence that semi-arid and desert soils sequester C in the aquifers and/or soils underneath in the form of inorganic C [101–103]. Desert soils in the Tarim Basin, for example, sequester inorganic C at a rate of 21.4 g C m^−2^ yr^−1^ in the aquifers C [101]. Inorganic C sequestration should not be neglected while counting terrestrial C sequestration in China. Attention in the future should be paid to the *in situ* measurements across a wider domain of semi-arid and desert soils, so as to robustly estimate inorganic C sequestration at a national scale.

More recent investigation shows that soil amendment with powdered basalt in natural ecosystems can remove atmospheric CO_2_ with great potential, though its side effects are unknown [104]. The basalt soil amendment should be considered as a possible option when assessing NCS options for offsetting anthropogenic CO_2_ emissions, but the feasibility and its effectiveness in China should be demonstrated first.

We focus on technologically achievable C sequestration via NCS in China, while full implementation of all cost-effective NCS worldwide, including 20 pathways of conservation, restoration and improved land management, can offer 37% of the needed mitigation through to 2030 and 20% through to 2050 [8]. To evaluate the future contribution of NCS to C reduction and C neutrality, it is required to quantify the maximum effectiveness of all available cost-effective NCS in China, such as the conservation of existing forests and improvements in plantations, cropland nutrient management, grazing-optimal intensity and grazing-animal management, and wetland conservation and restoration. The implementation of NCS in China is no doubt a viable option in accomplishing carbon neutrality. It is also important to act on the full range of possible incentives and policy levers [105]. Launching action now and learning from past experience can help deliver climate mitigation and sustainable development goals [106].

## METHODS

### Literature survey

We screened and reviewed peer-reviewed journal articles published prior to the end of June 2021 in Web of Science, Google Scholar and China Knowledge Resource Integrated Database (CNKI) to obtain the data associated with terrestrial C sequestration in China. Carbon sequestrations in different ecosystems were synthesized and analysed using these data

(Supplementary Methods). All of these data meet the following criteria: (i) the period and the area of investigation are clear for a given ecosystem; (ii) the duration of given management practice, such as exclosure of degraded grassland from grazing, is clear; (iii) the depth of soil sampling is clear; and (iv) filed measurements have replicates. When necessary, the raw data were extracted by digitizing graphs using the GetData Graph Digitizer v. 2.24 (free software at http://getdata-graph-digitizer.com/). Table S10 shows a summary of variables in the existing estimates and site-specific observations with necessary information.

### SOC sequestration vs ecosystem C sequestration

Terrestrial C sequestration includes C in both vegetation and soils. Due to intensive labor work in the measurement of SOC *in situ*, the ratio of soil to ecosystem C sink is usually used to estimate soil C sequestration. Approximately 30% of forest C sequestration is attributed to soils in Europe [107] and 33% in Russian forests [108]. These ratios have not been verified in China, though they were adopted to estimate soil C sequestration in China's [109] and the world's forests [110].

Synthesizing the surveyed data, we grouped the ratio of soil to ecosystem C sequestration for forests by latitude, but did not group for shrubland and grassland due to insufficient data (Supplementary Methods). The ratio of soil to ecosystem C sequestration was computed using Equation (1):

(1) \begin{equation*} {R_{{C_{Soil}}}} = \frac{{d{C_{Soil}}}}{{d{C_{ECO}}}}\ = \frac{{d{C_{Soil}}}}{{d{C_{Soil}} + d{C_{VG}}}}, \end{equation*}

where ${R_{{C_{Soil}}}}$ is the ratio of soil to ecosystem C sequestration. $d{C_{ECO}}$, $d{C_{Soil}}$ and $d{C_{VG}}$ represent the changes in ecosystem C, SOC and vegetation C in a given period, respectively.

The soil C sequestration (*C_Soil_*) can be computed using Equation (2) when the vegetation C sequestration (*C_VG_*) is available. The vegetation C sequestration can also be estimated using Equation (3) when *C_Soil_* is available:

(2) \begin{equation*} {C_{Soil}} = \frac{{{R_{{C_{Soil}}}}}}{{1 - {R_{{C_{Soil}}}}}}\ \times {C_{VG}}, \end{equation*}

(3) \begin{equation*} {C_{VG}} = \frac{{1 - {R_{{C_{Soil}}}}}}{{{R_{{C_{Soil}}}}}}\ \times {C_{Soil}}, \end{equation*}

where *C_VG_* and *C_Soil_* represent C sequestration in vegetation and soil without CO_2_ fertilization, respectively. We did not regard the increase in the above-ground biomass (AGB) in grassland, cropland and wetland as an acceptable C sink because the AGB in these ecosystems is generally used as livestock food (i.e. grazing and hay), falls into the surface as litter or is harvested (Supplementary Methods).

### CO_2_ fertilization effect

CO_2_ fertilization has led to a large increase in the land C uptake in the recent past [29–31]. The percentage contribution of CO_2_ fertilization to land C uptake ranges from 33% to 85% with a mean of 55% [61]. The CO_2_ fertilization effect on GPP was found to be 0.13% ppm^–1^ for high-latitude ecosystems (60°N–90°N) and 0.11% ppm^–1^ for extra-tropical ecosystems (30°N–90°N) [63]. Similarly, the CO_2_ fertilization effect on global GPP was estimated to be 0.138% ppm^−1^ [31]. These two estimates are comparable with the response of NPP to elevated CO_2_ concentrations (0.128 ± 0.001% ppm^–1^) in four free-air CO_2_ enrichment experiments in forest stands (35°54’N–45°40’N) [64]. We used the value of 0.13% ppm^–1^ to quantify the CO_2_ fertilization effect as:

(4) \begin{equation*} {f_{C{O_2}}} = \ 1 + 0.0013 \times \left( {\left[ {C{O_2}{]_i} - } \right[C{O_2}{]_{2010}}}\right), \end{equation*}

where ${f_{C{O_2}}}$ is the CO_2_ fertilization effect. [*CO*_2_]*_i_* is the atmospheric CO_2_ concentration (ppm) in the *i*th year (*i* = 2010, …, 2060) under RCP2.6 and RCP4.5 scenarios. [*CO*_2_]_2010_ is the atmospheric [*CO*_2_] in 2010. We used the observed atmospheric [*CO*_2_] between 2010 and 2019. We did not take RCP6.0 and RCP8.5 scenarios into consideration because global efforts are reducing anthropogenic CO_2_ emissions. The vegetation and soil C sequestration in the *i*th year (*i* = 2010, …, 2060) with CO_2_ fertilization ($C_{VG,\ i}^{C{O_2}},\ C_{Soil,\ i}^{C{O_2}}$) in a given ecosystem were then calculated using Equations (5) and (6), respectively:

(5) \begin{equation*}C_{VG,i}^{C{O_2}} = \ \left( {{C_{V{G_{B,i}}}} + \Delta {C_{VG,i}}} \right) \times {f_{C{O_2}}}, \end{equation*}

(6) \begin{eqnarray*} C_{Soil, i}^{C{O_2}} =\\ \left\{\begin{array}{@{}*{1}{l}@{}} {\frac{{{R_{{C_{Soil}}}}}}{{1 - {R_{{C_{Soil}}}}}} \times C_{VG, i}^{C{O_2}}\ \textit{for forest and shrubland}}\\ {}\\ {\left( {{C_{Soi{l_{B,i}}}} + \Delta {C_{Soil,i}}} \right)}\\ {\quad \times {f_{C{O_2}}}\ \textit {for grassland and wetland}}, \end{array}\right. \end{eqnarray*}

(7) \begin{equation*} {\rm{CSR}}_{i,j} = C_{VG, i}^{C{O_2}}\ + C_{Soil, i}^{C{O_2}}, \end{equation*}

where ${C_{V{G_{B, i\ }}}}$represents the vegetation C sequestration at baseline. $\Delta {C_{VG, i}}$ and $\Delta {C_{Soil,i}}$ are the increments of vegetation and soil C sequestration in the

*i*th year (*i* = 2010, …, 2060) under TOMs, respectively. ${\rm{CSR}}_{i,j}$ is the C sequestration rate in the *i*th year for a given ecosystem *j*. Vegetation C sequestration in croplands and wetlands is not included in ${\rm{CSR}}_{i,j}$. The calculations of $\Delta {C_{VG, i}}$ for forest, shrubland and grassland are given in Equations (S2), (S3) and (S5) in the Supplementary Methods. The Supplementary Methods also show the calculations of $\Delta {C_{Soil,i}}$ for grassland and wetland in Equations (S7) and (S9), respectively. Carbon sequestration in cropland soils was computed using a biogeophysical model (Agro-C). Agro-C [112] has been widely tested in China and was used to compile a national inventory of SOC change in agricultural soils in the People's Republic of China Third National Communication on Climate Change [5]. Detailed methods and data sources are described in the Supplementary Methods.

The total amount of C sequestration (${\rm{TCS}}{_i}$) in five terrestrial ecosystems in the *i*th year (*i* = 2010, …, 2060) was calculated using:

(8) \begin{equation*} {\rm{TCS}}{_i} = \mathop \sum \limits_{j = 1}^5 {\rm{CSR}}{_{i,j}}, \end{equation*}

where subscript *j* represents a given ecosystem. The uncertainty of the C sequestration estimates in all ecosystems was computed using an aggregated standard deviation (${\rm{SD}}{_i}$) as:

(9) \begin{equation*}{\rm{SD}}{_i} = \sqrt {\mathop \sum \limits_{j = 1}^5 SD_{i,j}^2}, \end{equation*}

where ${\rm{SD}}{_i}$is the aggregated standard deviation in the *i*th year (*i* = 2010, …, 2060). Subscript *j* represents a given ecosystem. $S{D_{i,j}}$ was derived from individual findings for a given ecosystem.

### Scenarios for TOMs

Net-zero CO_2_ emissions implies a balance between anthropogenic CO_2_ emissions and removals over a specified period [113]. NCS actions include protection, restoration and sustainable management of natural C sinks and reservoirs that increase C storage and/or avoid greenhouse gas emissions across global forests, wetlands, grasslands and agricultural lands [8]. The forest area in China was 98.08 Mha during 1950−1962 [37] and increased to 220.6 Mha in 2020, projected to be 267.6 Mha in 2050 under TOM [45]. The increase in forest area was and should be attributed to reforestation. Furthermore, C sequestration in natural forests is also included in national greenhouse gas inventories [77].

Following NCS actions, the TOMs include the expansion of forest areas, restoration of moderately and above degraded grasslands via exclosure from grazing, increase in the proportion of crop residue retention and no-till areas, and restoration of wetland (Table 3).

### Calculating the contribution of terrestrial C sequestration to offsetting energy-related CO_2_ emissions

The contribution of terrestrial C sequestration to offsetting energy-related CO_2_ emissions was calculated using Equation (10):

(10) \begin{equation*} P_{\rm{ECO}{_2}}^{\rm{TCS}}\left( \% \right) = \left\{ \begin{array}{@{}*{1}{c}@{}} {\frac{{\rm{TCS}{_i}}}{{\rm{ECO}{_{2, i}}}}\ {\rm{for}}\ i = \ 2010\ to\ 2030}\\ {}\\ {\frac{{\rm{TCS}{_i}}}{{\rm{ECO}{_{2, 2030}}}}\ {\rm{for}}\ i = \ 2031\ to\ 2060}, \end{array}\right. \end{equation*}

where $P_{\rm{ECO}{_2}}^{\rm{TCS}}( \% )$ is the proportion of terrestrial C sequestration offsetting energy-related CO_2_ emissions. ${\rm{TCS}}{_i}\ $is the total amount of C sequestration in five terrestrial ecosystems in the *i*th year under different scenarios. The C sequestration was converted to CO_2_ by a coefficient of 44/12. ${\rm{ECO}}{_{2, i}}$ and ${\rm{ECO}}{_{2, 2030}}$ represent energy-related CO_2_ emissions in the *i*th year and the year 2030, respectively. The People's Republic of China Third National Communication on Climate Change [5] reports energy-related CO_2_ emissions in 2010, 2020 and 2030 under different policy scenarios. The annual energy-related CO_2_ emissions before 2030 were linearly interpolated between 2010 and 2020, and between 2020 and 2030.

## DATA AVAILABILITY

China's terrestrial C sequestration data in different ecosystems reported in this study, together with methods and data sources are included in Supplementary Data.

## Supplementary Material

nwac057_Supplemental_File
