# Supplementary material for: The role of China's terrestrial carbon sequestration 2010–2060 in offsetting energy-related CO2 emissions
Source: Natl Sci Rev. 2022 Mar 25;9(8):nwac057. doi: 10.1093/nsr/nwac057 (PMC9385465; doi:10.1093/nsr/nwac057)
Supplement: nwac057_Supplemental_File [file nwac057_supplemental_file.pdf]

Supplementary Information for

The role of China's terrestrial carbon sequestration 2010–2060 in offsetting energy-related CO_2 emissions

Yao Huang[1,2,*,#], Wenjuan Sun[1,#], Zhangcai Qin[3], Wen Zhang[2], Yongqiang Yu[2], Tingting Li[2], Qing Zhang[2], Guocheng Wang[2], Lingfei Yu[1], Yijie Wang[1], Fan Ding[4], Ping Zhang[5]

[1] State Key Laboratory of Vegetation and Environmental Change, Institute of Botany, Chinese Academy of Sciences, Beijing 100093, China

[2] State Key Laboratory of Atmospheric Boundary Layer Physics and Atmospheric Chemistry, Institute of Atmospheric Physics, Chinese Academy of Sciences, Beijing 100029, China

[3] School of Atmospheric Sciences, Sun Yat-sen University, Guangzhou 510275, China

[4] College of Land and Environment, Shenyang Agricultural University, Shenyang 110866, China

[5] College of New Energy and Environment, Jilin University, Changchun 130021, China

[*]Corresponding author. E-mail: huangyao@ibcas.ac.cn

ORCID: https://orcid.org/0000-0002-0192-1421

[#]Equally contributed to this work.

Supplementary details in terrestrial C sequestration in different ecosystems

China's terrestrial ecosystems can sequester 0.375±0.056 Pg C yr^{-1} (1 Pg $=10^{12}$ kg) in the 2010s and increase to 0.458±0.100 Pg C yr^{-1} in the 2050s under RCP2.6 scenario, and to 0.493±0.108 Pg C yr^{-1} under RCP4.5 scenario (Table S1).

Forest dominates terrestrial C sequestration, contributing 67.8–71.4% to the total amount. The C sequestration rate was estimated to be 259.5 Tg C yr^{-1} under both RCP2.6 and RCP4.5 scenarios in the 2010s, but it can increase to 327.0 Tg C yr^{-1} under RCP2.6 and 349.6 Tg C yr^{-1} under RCP4.5 scenario in the 2050s (Table S2). The increase rates are 17.4 and 23.1 Tg C $decade^{-1}$ under RCP2.6 and RCP4.5 scenarios, respectively.

Shrubland can sequester approximately 28 Tg C yr^{-1} at baseline, but the C sequestration will sharply decrease to approximately 11 Tg C yr^{-1} in the 2050s (Table S3) due to a reduction of area under target-oriented managements (TOMs). The decrease rates are 3.3 and 3.1 Tg C $decade^{-1}$ under RCP2.6 and RCP4.5 scenarios, respectively. Carbon sequestration in grassland can be 18.0 Tg C yr^{-1} in the 2010s, and increase to 27.5–30.5 Tg C yr^{-1} in the 2050s. Part of this increase is attributed to the TOMs implementation, with contribution of 13.9% in the 2020s and 30.5% in the 2050s to the total C sequestration (Table S4).

Cropland soils can sequester C at a rate of 43.1 Tg C yr^{-1} in the 2010s, and 52.6–58.4 Tg C yr^{-1} in the 2050s (Table S5). Crop residue retention and the expansion of no-till management play a key role in improving C sequestration, contributing a total of 10.8% in the 2010s and 48.6% in the 2050s to respective C sequestration. Wetland soils can sequester 30.5–42.8 Tg C yr^{-1} between 2010s–2050s (Table S6), 2.7–21.7% of which is attributed to wetland restoration.

Supplementary Methods

Scenarios

Climate. The CO_2 concentrations in RCP2.6 and RCP4.5 scenarios were used to compute CO_2 fertilization effect. We used the actual CO_2 concentration between 2010–2019. The CO_2 concentrations in RCP8.5 scenario was not taken into consideration since global efforts are making to reduce CO_2 emissions to achieve Paris Agreement carbon targets.

Energy CO_2 emissions. The People's Republic of China Third National Communication on Climate Change projected the trends of future CO_2 emissions from energy and industrial processes under different scenario assumptions [1]. We pay specific attention to energy-related CO_2 emissions since energy consumption accounts for approximately 88% of the total CO_2 emissions in China. Three scenarios with different assumptions were documented in the People's Republic of China Third National Communication on Climate Change [1], namely reference scenario, policy scenario I and policy scenario II (Table S7).

Reference scenario: It takes economic policies (promoting economic transformation and upgradation) and spontaneous improvement in energy efficiency into consideration, but without strict control and restraint on future carbon emissions.

Policy scenario I: It takes economic policies (promoting economic transformation and upgradation) and strict control and restraint on future carbon emissions into consideration. The future carbon intensity (CO_2 emission per unit GDP) is assumed to decrease by 4–5% annually.

Policy scenario II: It takes economic policies (promoting economic transformation and upgradation) and strict control and restraint on future carbon emissions into consideration. The future carbon intensity is assumed to decrease by 5–6% annually.

Estimation of terrestrial C sequestration in different ecosystems

Forest C sequestration

Baseline. The area in 2010 is designed as reference. Actual forest area is adopted for 2010–2020, and the rear remains unchanged after 2020.

Target-oriented managements. In the light of the Forestry Action Plan to Address Climate Change [9], the forest area in 2050 is proposed to increase by 47 million hectors based on the 2020 value. We assumed a linear increase in the forest area from 2021 to 2060 (Fig. S1). 60% of this increased area comes from shrubland and 40% from grassland (Table S8).

Vegetation C sequestration. We used available estimates of future C sequestration in above-ground biomass (AGB) [10–12] to quantify yearly C sequestration by taking forest area and CO_2 fertilization into consideration. Carbon sequestration in below-ground biomass (BGB) was calculated using a nationwide mean value of root to shoot ratio [24]. Analyzing the literature survey data [12–26] that covers the period from the mid-1970s to 2010s, we found that there exists a robust linear relationship between the AGB C pool C_{AGB}^{Pool} (Pg C) and forest area S_{forest} (million hectare) (Fig. S3a) as:

$$C_{AGB}^{Pool} = 0.032 \times S_{forest} + 0.75 \quad (R^2 = 0.85, n = 71, p < 0.001) \tag{S1}$$

The slope of Eqn. (S1) suggests that the vegetation C pool can increase by 320 kg C ha^{-1} with an increase of one hectare in forest area. Taking the forest area and vegetation C pool in 2010 as reference, we determined the correction coefficient of vegetation C sequestration in relation to the forest area (Fig. S1) over the period 2010–2060 (Fig. S3b). The estimates of future vegetation C sequestration [10–12] were then corrected to a unified forest area (Fig. S1a). Using Eqn. (3) in the main text, we also estimated the vegetation C sequestration in the light of soil C sequestration [27] and made correction to a unified forest area.

The increment of vegetation C sequestration under TOM ($\Delta C_{VG,i}^{forest}$) was computed by:

$$\Delta C_{VG,i}^{forest} = 320 \times (1 + R_{r:s}) \times (S_{forest,i+1} - S_{forest,i}) \tag{S2}$$

where $R_{r:s}$ is the root to shoot ratio. $S_{forest,i}$ is forest area (hectare) in the ith year (i =2010, …, 2060).

Soil C sequestration. Analyzing the literature survey data [28–81], we found that the ratios of soil C sequestration to ecosystem C sequestration ($R_{C_{Soil}}$) in forest are latitude-related (Table S9). In the light of provincial forest area, we determined an area-weighted ratio of 0.276. Using Eqn. (2) in the main text, the yearly soil C sequestration was computed.

Shrubland C sequestration

Baseline. The area in 2010 is designed as reference, and remains unchanged after 2010.

Target-oriented managements. Part of the shrubland area is assumed to be converted to forest. The area of shrubland was 74.3 Mha in 2010 [24], and will linearly decrease to 24.73 Mha in 2060 (Table S8).

Vegetation and soil C sequestration. Very few investigations have focused on C sequestration in shrubland at national scale so far, especially for the future estimates. Using available estimates across 1980s–2000s [82,83] as reference, we computed yearly vegetation C sequestration by extrapolating these estimates to 2010–2060 in the light of area change

(Table S8). The vegetation C sequestration includes above-ground and below-ground biomass [82, 83]. According to the shrubland SOC density in the 1980s [84] and in the 2000s [85], we estimated shrubland soil C sequestration. Similar to the calculation of yearly vegetation C sequestration, we estimated the yearly SOC sequestration by taking the changes in SOC density between the 1980s and the 2000s as reference. Using Eqn. (3) in the main text, the yearly vegetation C sequestration was computed; The yearly SOC sequestration was also estimated in the light of yearly vegetation C sequestration by using Eqn. (2) in the main text. The ratio of soil to ecosystem C sequestration is 0.645 in shrubland [82].

The increment of vegetation C sequestration under TOMs ($\Delta C_{VG,i}^{shrub}$) was calculated by:

$$\Delta C_{VG,i}^{shrub} = 126.5 \times (S_{shrub,i+1} - S_{shrub,i}) \quad \text{(S3)}$$

where $S_{shrub,i}$ is shrubland area (hectare) in the ith year (i =2010, …, 2060). Constant 126.5 is a mean rate of vegetation C sequestration (kg C ha^{-1} $year^{-1}$) during 1980s–2000s, derived from the literature [82,83] and calculated from soil C sequestration using Eqn. (3) in the main text.

Grassland

Baseline. The area in 2010 is designed as reference, and remains unchanged after 2010.

Target-oriented managements. Approximately 90% of grassland in China shows signs of deterioration, of which moderately and severely degraded grassland is 60% [86]. Grazing exclusion (GE) is well recognized to be an important strategy for restoring degraded grasslands and promoting C storage [87]. The improvements in hay production in China's grassland is substantially attributed to GE (Fig. S2). Grasslands in Tibet, Inner Mongolia, Xinjiang, Qinghai and Gansu Province account for approximately 70% of the total grassland area in China. We assumed that 50% of the moderately and above degraded area (57.5 Mha) in these regions will be restored by exclosure from grazing between 2021 and 2060, and the yearly area of enclosure increases linearly.

Below-ground biomass C sequestration. We did not regard the increase in above-ground biomass (AGB) as an acceptable carbon sink but the changes in BGB count, because AGB is generally used as livestock food (i.e., grazing and hay) or fallen into the surface as litters. Assuming a constant ratio of AGB to BGB for each grassland type, the BGB C sequestration was set to be 18.1 kg C ha^{-1} yr^{-1} [88] at the baseline.

Analyzing the literature survey data [89–99], we found that the relative changes in the BGB C can be fitted by a non-linear function against the years since adopting exclosure from grazing (Fig. S4) as:

$$R_{BGB,i}^{GE} = \exp\left(1.059 - 0.07 \times i - \frac{5.116}{i}\right) \quad (R^2 = 0.19, n = 20, p < 0.05) \quad \text{(S4)}$$

where $R_{BGB,i}^{GE}$ is a relative change in BGB C defined as $BGB_{GE,i}/BGB_{DG}$. The $BGB_{GE,i}$ and BGB_{DG} represent BGB C under grazing exclusion and corresponding degraded grassland, respectively. i is the years since exclosure from grazing. Analyzing the data from a review work [100], we determined that an average ratio of BGB_{DG} to BGB_{GE} is 0.48.

The increment of BGB C sequestration in the ith year since adopting GE ($\Delta C_{BGB_i}^{grass}$) was then computed by:

$$\Delta C_{BGB_i}^{grass} = 0.48 \times 18.1 \times R_{BGB,i}^{GE} \times S_{grass}^{GE} \quad \text{(S5)}$$

where constant 18.1 is the BGB C sequestration at the baseline. S_{grass}^{GE} is the area (hectare) in a given year when grazing exclusion begins.

A synthesis of the effect of grazing exclusion on carbon dynamics in China's grasslands shows that the rates of increase in BGB and soil C content keep relatively stable after 15 years of GE [87]. We constrained the GE effect to 15 years, and the BGB C sequestration after 15 years since GE will follow the sequestration rate in the 15th year.

Soil C sequestration. Based on available estimates of soil C sequestration in grassland [82, 101–103], we used a mean rate of 36.4 kg C ha^{-1} yr^{-1} to compute soil C sequestration at baseline.

Using the literature survey data [104–108], we determined a nonlinear function (Fig. S5a) to simulate the changes in SOC under exclosure from grazing as:

$$R_{SOC,i}^{GE} = 2.133 + \exp(2.332 - 0.188 \times i) \quad (R^2 = 0.71, n = 15, p < 0.001) \quad \text{(S6)}$$

where $R_{SOC,i}^{GE}$ is a relative change in SOC density defined as $SOCD_{GE,\,i}/SOCD_{DG}$ (% yr^{-1}). The $SOCD_{GE,i}$ and $SOCD_{DG}$ represent SOC density under grazing exclusion and corresponding degraded grassland, respectively. i is the years since exclosure from grazing.

The increment of soil C sequestration in the ith year since adopting GE ($\Delta C_{Soil_i}^{grass}$) was then computed by:

$$\Delta C_{Soil_i}^{grass} = 0.5 \times SOCD_{UDG} \times (0.01 \times R_{SOC,i}^{GE}) \times S_{grass}^{GE} \quad \text{(S7)}$$

where $SOCD_{UDG}$ represents the SOC density in undegraded grassland. S_{grass}^{GE} is the area (hectare) in a given year when grazing exclusion begins. Analyzing the data from a review work [100], the $SOCD_{UDG}$ was determined to be 56.3 Mg C ha^{-1} (0-30cm depth), and an average ratio of $SOCD_{DG}$ to $SOCD_{UDG}$ was determined to be 0.50. Similar to the computation of BGB C sequestration under grazing exclusion, we constrained the GE effect to 15 years. The SOC sequestration after 15 years since GE will follow the sequestration rate in the 15th year. The accumulated increase in SOCD under grazing exclusion in the 15th year will reach 80% of that in undegraded grassland (Fig. S5b).

Cropland

Above-ground biomass in cropland is subsequently harvested and used, releasing CO_2 back to the atmosphere within less than a year [109]. We thus did not regard the increase in AGB as an acceptable carbon sink.

Baseline. The area remains unchanged. Crop yield, the proportion of crop residue retention, manure input and no-till practice remain at the 2010s level.

Target-oriented managements. Part of the area will be transformed to wetland after 2020. Crop yield will increase by 0.5% per year between 2021 and 2060 based on the mean of 2016–2020 (https://data.stats.gov.cn/). The proportion of crop residue retention will increase from the baseline to 60% in 2060, and manure input will remain unchanged. The proportion of no-till area will increase from the baseline to 30% in 2060. The yearly changes in cropland area, the proportion of crop residue retention and no-till area was assumed to be linear.
Soil C sequestration. A biogeophysical model (Agro-C) for simulating the carbon budget of agroecosystems was used to simulate the changes in SOC at the baseline and the target-oriented management.

Agro-C model consists of two submodels: Crop-C for simulating crop NPP, and Soil-C for computing SOC [110]. Crop-C simulates crop photosynthesis and autotrophic respiration. Soil-C simulates the decomposition of organic carbon in soils with a first-order kinetics reaction. Changes in SOC are determined by a balance between the loss of soil carbon and the sequestration of input organic carbon. We used Monte Carlo analysis to develop the probability density functions (PDF) for C input within the ranges of mean±10%. The variation of ±10% in C input was assigned as uncertainties of estimated SOC changes. The Agro-C model has been widely tested in China [110–112] and Australia [113], and was used to compile national inventory of SOC change in agricultural soils in The People's Republic of China Third National Communication on Climate Change [1]. Model input included projected climates under RCP2.6 and RCP4.5 scenarios and corresponding atmospheric CO_2 concentration, soil properties, and target-oriented management with 10 km×10 km resolution. The changes in SOC have the same resolution as model input.

Wetland
Baseline. We focused on three types of wetlands (inland marshes and swamp, coastal wetland, artificial wetland) and did not take rivers and lakes into consideration. The area of three types of wetlands remains unchanged as documented in the second survey of wetlands in China.
Target-oriented managements. In the light of the national plans and the periodical objectives [114, 115], the wetland area through restoration increased by 474.5×10^3 ha between 2010 and 2020, and will increase by 929.5×10^3 ha between 2021 and 2030 relative to 2020. The wetland restoration primarily includes the abandonment of cropland, re-wetting in freshwater wetlands, and plantation and irrigation in extreme degraded wetlands.
Soil C sequestration. Similar to grassland, we did not regard the increase in above-ground biomass (AGB) as an acceptable carbon sink. A review work shows the soil C sequestration rates along a latitudinal climate gradient [116]. In the light of provincial wetland area and climate zone, we determined an area-weighted C sequestration rate of 854 kg C ha^{-1} yr^{-1} using the data in the review work [116]. This area-weighted rate was used to compute soil C sequestration at baseline.

Analyzing the literature survey data [117–131], we determined a linear function (Fig. S6) of the changes in SOC density relative to control plots as:

$$R_{SOC,i}^{RS} = 0.079 \times i + 0.86 \quad (R^2 = 0.80, n = 9, p < 0.01) \tag{S8}$$

where $R_{SOC,i}^{RS}$ is a relative change in SOC density (0–30cm) defined as $SOCD_{RS}/SOCD_{CK}$.

The $SOCD_{RS}$ and $SOCD_{CK}$ represent SOC density in restored wetland and corresponding control, respectively. i is the years since restoration. The slope of Eqn. (S8) suggests that the SOC sequestration in the restored wetland can increase by 7.9% per year relative to the SOC in the control.

The increment of soil C sequestration in the ith year since restoration ($\Delta C_{Soil_i}^{wetland}$) is then computed as:

$$\Delta C_{Soil_i}^{wetland} = SOCD_{CK} \times 0.079 \times i \times S_{wetland}^{RS} \quad \text{(S9)}$$

where $S_{wetland}^{RE}$ is the area (hectare) of wetland restoration in a given year, and i is the years since restoration. We assumed that $SOCD_{CK}$ is the current cropland's SOC density (37.4 Mg C ha^{-1}) in the 0–30 cm depth [132]. Eqn. (S9) suggests that the restored wetland SOC sequestration in the 15th year since restoration is 2.2-fold as much as in the control. We further assumed that the soil C sequestration after 15 years since restoration will follow the sequestration rate in natural wetlands since the SOCD (0-20cm) in wetland soils is about twice as in cropland soils [102].

Table S10 shows a summary of variables in the existing estimates and site-specific observations with corresponding reference and necessary information.

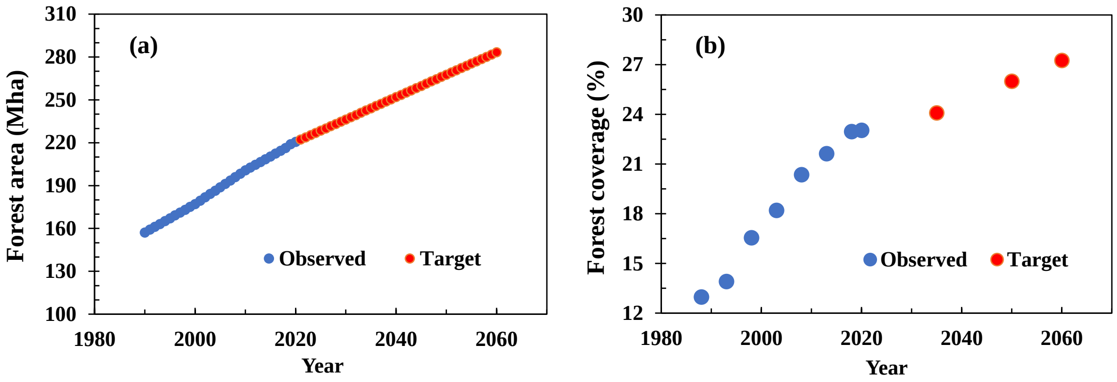

Fig. S1. Observed and target forest area (a) and forest coverage (b) in China.

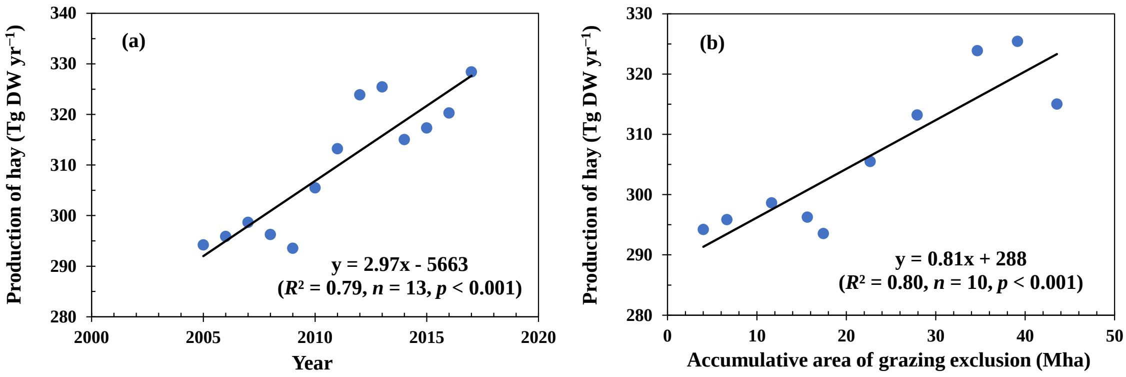

Fig. S2. Changes in the production of hay from 2005 to 2017 in China's grasslands (a), and the correlation of hay production (2005−2014) with the accumulative area of grazing exclusion (2003−2012) (b). The hay production has 2-year lags with the accumulative area of grazing exclusion. Data from yearly China Environmental Status Bulletin (2003−2017). The areas of grazing exclusion from 2013 to 2017 are not available.

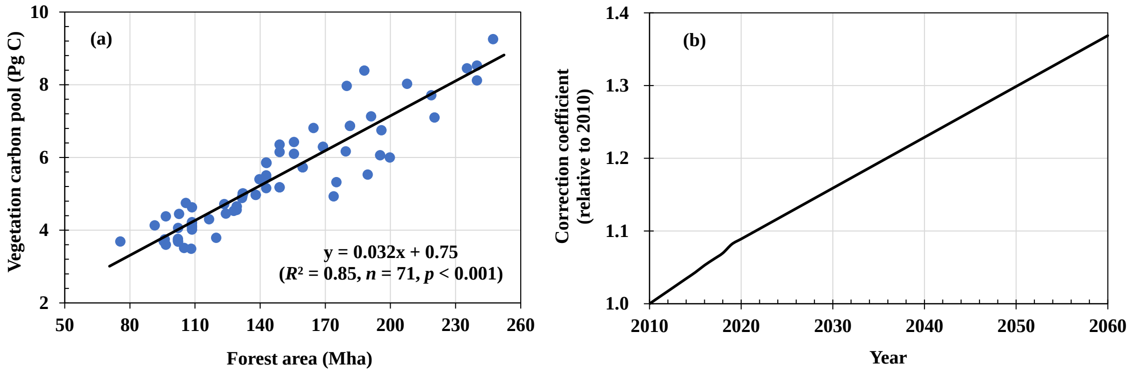

Fig. S3. Relationship between vegetation carbon pool and area in forest (a), and correction coefficient relative to 2010 forest area (b).

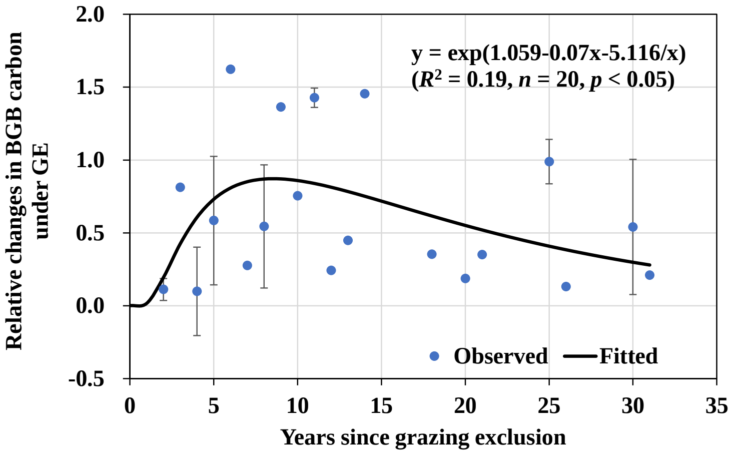

Fig. S4. Non-linear fitting for the changes in belowground biomass (BGB) carbon under grazing exclusion (GE). Vertical bars show standard errors.

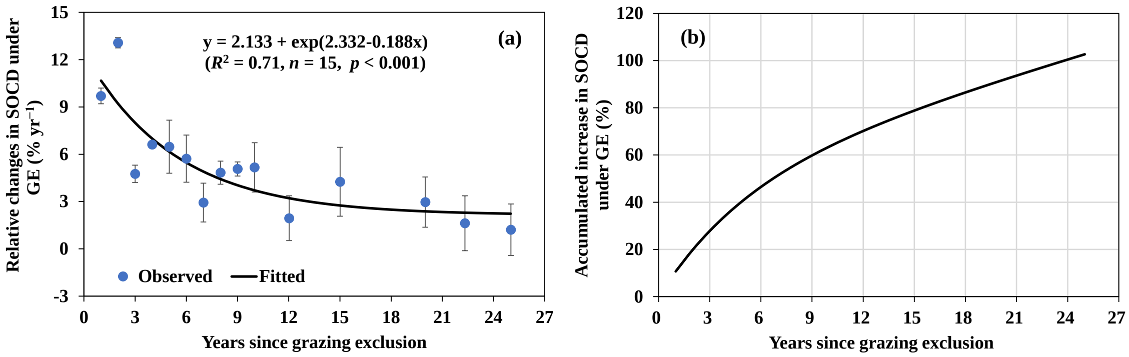

Fig. S5. Non-linear fitting for the changes in soil organic carbon density (SOCD) (a), and accumulated increase in SOCD (b) under grazing exclusion (GE). Vertical bars show standard errors.

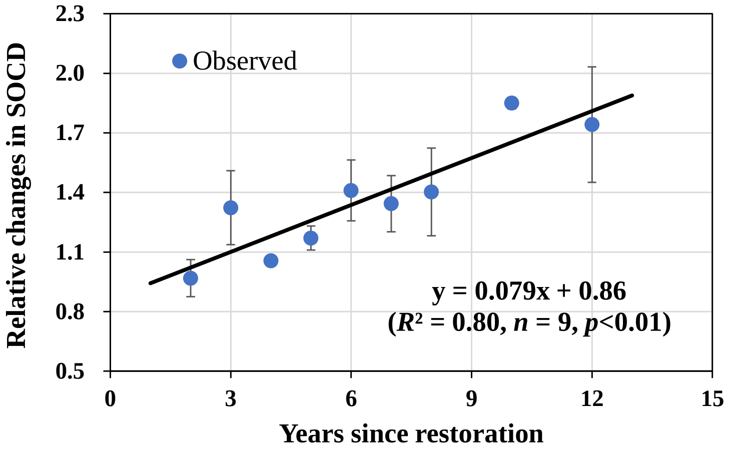

Fig. S6. Changes in wetland soil organic carbon. Vertical bars show standard errors.

Table S1. Estimated carbon sequestration rate (Pg C yr^{-1}) in China's terrestrial ecosystems at baseline and target-oriented managements under two climate scenarios.

Component	Decade	RCP2.6					RCP4.5				
		Baseline	TOMs[a]	Subtotal	SD	%[b]	Baseline	TOMs	Subtotal	SD	%
Vegetation	2010s	0.195	0.008	0.203	0.027	4.0	0.195	0.008	0.203	0.027	4.0
	2020s	0.188	0.022	0.210	0.029	10.5	0.188	0.022	0.210	0.029	10.5
	2030s	0.189	0.036	0.225	0.032	15.8	0.193	0.036	0.229	0.032	15.8
	2040s	0.189	0.048	0.237	0.050	20.1	0.197	0.050	0.247	0.052	20.1
	2050s	0.191	0.059	0.250	0.061	23.7	0.204	0.063	0.267	0.065	23.8
Soil	2010s	0.166	0.007	0.173	0.029	4.1	0.166	0.007	0.173	0.029	4.1
	2020s	0.162	0.025	0.187	0.033	13.4	0.162	0.025	0.187	0.033	13.4
	2030s	0.162	0.041	0.203	0.035	20.3	0.165	0.042	0.207	0.036	20.4
	2040s	0.161	0.046	0.207	0.038	22.3	0.168	0.049	0.217	0.040	22.4
	2050s	0.160	0.049	0.209	0.039	23.4	0.173	0.053	0.226	0.043	23.6
Total	2010s	0.361	0.015	0.375	0.056	4.0	0.361	0.015	0.375	0.056	4.0
	2020s	0.350	0.047	0.397	0.062	11.9	0.351	0.047	0.397	0.062	11.9
	2030s	0.351	0.077	0.428	0.067	17.9	0.358	0.078	0.435	0.068	17.9
	2040s	0.350	0.094	0.444	0.088	21.1	0.366	0.098	0.464	0.092	21.2
	2050s	0.351	0.108	0.458	0.100	23.6	0.377	0.117	0.493	0.108	23.7

[a] Target-oriented managements. [b] the ratio of TOMs to subtotal.

Table S2. Estimated carbon sequestration rate (Tg C yr^{-1}) at baseline and target-oriented managements under two climate scenarios – Forest

Component	Decade	RCP2.6				RCP4.5			
		Baseline	TOMs[a]	Subtotal	%[b]	Baseline	TOMs	Subtotal	%
Vegetation	2010s	179.2	8.7	187.9	4.6	179.2	8.7	187.9	4.6
	2020s	172.9	22.1	195.0	11.3	173.2	22.1	195.3	11.3
	2030s	175.9	34.6	210.5	16.5	178.4	35.2	213.8	16.5
	2040s	175.9	47.2	223.1	21.1	183.0	49.1	232.1	21.2
	2050s	177.0	59.7	236.7	25.2	189.3	63.8	253.1	25.2
Soil	2010s	68.5	3.3	71.7	4.6	68.5	3.3	71.7	4.6
	2020s	66.0	8.5	74.2	11.4	66.0	8.5	74.5	11.4
	2030s	67.1	13.4	80.2	16.7	67.9	13.4	81.5	16.4
	2040s	67.1	18.0	85.1	21.2	69.8	18.5	88.4	21.0
	2050s	67.4	22.6	90.3	25.1	72.3	24.3	96.5	25.1
Total	2010s	247.6	12.0	259.5	4.6	247.6	12.0	259.5	4.6
	2020s	238.9	30.5	269.2	11.3	239.2	30.5	269.5	11.3
	2030s	242.7	48.0	290.7	16.5	246.5	48.8	295.1	16.5
	2040s	243.0	64.9	308.2	21.1	252.8	67.6	320.7	21.1
	2050s	244.6	82.4	327.0	25.2	261.5	88.1	349.6	25.2

[a] Target-oriented managements. [b] the ratio of TOMs to subtotal.

Table S3. Estimated carbon sequestration rate (Tg C yr^{-1}) at baseline and target-oriented managements under two climate scenarios – Shrubland

Component	Decade	RCP2.6				RCP4.5			
		Baseline	TOMs[a]	Subtotal	%[b]	Baseline	TOMs	Subtotal	%
Vegetation	2010s	9.5	-0.8	8.7	-9.4	9.5	-0.8	8.7	-9.4
	2020s	9.8	-2.2	7.6	-28.6	9.8	-2.2	7.6	-28.6
	2030s	9.8	-3.5	6.3	-56.5	10.1	-3.5	6.5	-54.2
	2040s	10.1	-4.9	5.2	-94.7	10.4	-4.9	5.5	-90.0
	2050s	10.1	-6.0	3.8	-157.1	10.6	-6.5	4.1	-160.0
Soil	2010s	17.2	-1.6	15.8	-10.3	17.2	-1.6	15.8	-10.3
	2020s	17.7	-4.1	13.6	-30.0	17.7	-4.1	13.6	-30.0
	2030s	18.0	-6.5	11.5	-57.1	18.3	-6.5	11.7	-55.8
	2040s	18.3	-8.7	9.3	-94.1	18.8	-9.0	9.8	-91.7
	2050s	18.3	-11.2	7.1	-157.7	19.4	-11.7	7.6	-153.6
Total	2010s	26.7	-2.5	24.3	-10.1	26.7	-2.5	24.3	-10.1
	2020s	27.5	-6.3	21.3	-29.5	27.5	-6.3	21.3	-29.5
	2030s	28.1	-10.1	18.0	-56.1	28.4	-10.1	18.3	-55.2
	2040s	28.1	-13.6	14.5	-94.3	29.2	-14.2	15.0	-94.5
	2050s	28.1	-17.2	10.9	-157.5	30.0	-18.3	11.7	-155.8

[a] Target-oriented managements. [b] the ratio of TOMs to subtotal.

Table S4. Estimated carbon sequestration rate (Tg C yr^{-1}) at baseline and target-oriented managements under two climate scenarios – Grassland

Component	Decade	RCP2.6				RCP4.5			
		Baseline	TOMs[a]	Subtotal	%[b]	Baseline	TOMs	Subtotal	%
Vegetation	2010s	6.0	0.0	6.0	0.0	6.0	0.0	6.0	0.0
	2020s	6.0	0.8	7.1	11.5	6.3	0.8	7.1	11.5
	2030s	6.3	1.9	8.2	23.3	6.5	1.9	8.2	23.3
	2040s	6.3	2.2	8.5	25.8	6.8	2.2	9.0	24.2
	2050s	6.3	2.5	8.7	28.1	7.1	2.7	9.8	27.8
Soil	2010s	12.0	0.0	12.3	0.0	12.0	0.0	12.3	0.0
	2020s	12.3	2.2	14.5	15.1	12.3	2.2	14.5	15.1
	2030s	12.5	4.4	17.2	25.4	12.8	4.6	17.5	26.6
	2040s	12.8	5.7	18.3	31.3	13.6	6.0	19.6	30.6
	2050s	12.8	6.0	18.8	31.9	14.2	6.5	20.7	31.6
Total	2010s	18.0	0.0	18.0	0.0	18.0	0.0	18.0	0.0
	2020s	18.5	3.0	21.5	13.9	18.5	3.0	21.5	13.9
	2030s	18.8	6.3	25.1	25.0	19.4	6.5	25.9	25.3
	2040s	19.1	7.6	26.7	28.6	20.5	8.2	28.6	28.6
	2050s	19.1	8.5	27.5	30.7	21.3	9.3	30.5	30.4

[a] Target-oriented managements. [b] the ratio of TOMs to subtotal.

Table S5. Estimated carbon sequestration rate (Tg C yr^{-1}) at baseline and target-oriented managements under two climate scenarios – Cropland

Component	Decade	RCP2.6				RCP4.5			
		Baseline	TOMs[a]	Subtotal	%[b]	Baseline	TOMs	Subtotal	%
Soil	2010s	38.2	4.6	43.1	10.8	38.2	4.6	43.1	10.8
	2020s	34.4	16.6	50.7	32.8	34.4	16.6	51.0	32.6
	2030s	30.5	26.5	57.0	46.4	31.4	27.0	58.4	46.3
	2040s	28.4	27.0	55.6	48.5	30.3	28.9	59.2	48.8
	2050s	27.0	25.6	52.6	48.7	30.0	28.4	58.4	48.6

[a] Target-oriented managements. [b] the ratio of TOMs to subtotal.

Table S6. Estimated carbon sequestration rate (Tg C yr^{-1}) at baseline and target-oriented managements under two climate scenarios – Wetland

Component	Decade	RCP2.6				RCP4.5			
		Baseline	TOMs[a]	Subtotal	%[b]	Baseline	TOMs	Subtotal	%
Soil	2010s	29.7	0.8	30.5	2.7	29.7	0.8	30.5	2.7
	2020s	30.5	3.3	34.1	9.6	30.5	3.3	34.1	9.6
	2030s	31.1	6.0	37.1	16.2	31.6	6.0	37.6	15.9
	2040s	31.4	7.4	38.7	19.0	32.5	7.6	40.1	19.0
	2050s	31.4	8.7	40.1	21.8	33.5	9.3	42.8	21.7

[a] Target-oriented managements. [b] the ratio of TOMs to subtotal.

Table S7. Projected CO_2 emission (Pg CO_2) from energy consumption ¶

Scenario	Year		
	2010	2020	2030
Reference scenario	7.6 (7.2–8.0)	10.8 (10.6–10.9)	12.5 (12.0–12.9)
Policy scenario-I		10.2 (9.8–10.5)	11.0 (10.5–11.5)
Policy scenario-II		9.8 (9.6–10.0)	10.2 (9.8–10.6)

¶ Values in 2010 are the national inventory report. Data come from the People's Republic of China Third National Communication on Climate Change (December 2018)

Table S8. Area of different ecosystems (10^6 ha)

Year	Forest	Shrubland	Grassland	Cropland	Wetland	Total
2010	200.61	74.30	331.00	130.00	34.27	770.2
2020	220.57	62.32	323.02	129.53	34.74	770.2
2030	236.23	52.92	316.75	128.60	35.67	770.2
2040	251.90	43.52	310.48	128.60	35.67	770.2
2050	267.57	34.13	304.22	128.60	35.67	770.2
2060	283.23	24.73	297.95	128.60	35.67	770.2

Table S9. Ratio of soil C sequestration to ecosystem C sequestration in forest

Item	Latitude			
	10ºS~10ºN	10~25ºS and 10~25ºN	25~35ºS and 25~35ºN	35~60ºN
n	6	41	50	69
Minimum	0.00	-0.05	-0.01	-0.06
Maximum	0.64	0.79	0.83	0.79
Mean	0.34	0.25	0.40	0.21
95% LCL	0.06	0.19	0.34	0.17
95% UCL	0.62	0.31	0.46	0.26

Table S10. Summary of variables in the existing estimates and site-specific observations with corresponding reference and necessary information

Forest	Existing estimates of C storage at national scale				Reference
	Year or period	AGB[a] (Tg C)	TB[b] (Tg C)	C_{SOCD}[c] (kg C ha^{-1} yr^{-1})	
	2000, 2010, 2020, 2030, 2040, 2050	√			[10]
	2010, 2020, 2030, 2040, 2050, 2060	√			[11]
	2003, 2008, 2013, 2020, 2050	√			[12]
	1980s–2000s			√	[27]
	1977–1981, 1984–1988, 1989–1993, 1994–1998	√			[13]
	1977–1981, 1984–1988, 1989–1993, 1994–1998, 1999–2003	√			[14]
	1977–1981, 1984–1988, 1989–1993, 1994–2003, 2004–2008	√			[15]
	1973–1976, 1984–1988, 1989–1993	√			[16]
	1973–1976, 1977–1981, 1984–1988, 1989–1993	√			[17]
	1989–1993	√			[18]
	1982–1999	√			[19]
	1973–1976, 1977–1981, 1984–1988, 1989–1993, 1994–1998, 1999–2003	√			[20]
	1989–1993, 1994–1998, 1999–2003	√			[21]
	1973–1976, 1977–1981, 1984–1988, 1989–1993, 1994–1998, 1999–2003, 2004–2008	√			[22]
	2004–2008	√			[23]
	1994–1998, 1999–2003, 2004–2008, 2010–2015	√			[24]
	2001–2005, 2006–2010	√			[25]
	1973–1976, 1977–1981, 1984–1988, 1989–1993, 1994–1998, 1999–2003, 2004–2008, 2009–2013, 2014–2018	√			[26]
**Shrubland**	1982–1999		√	√	[82]
	1982–1990, 1991–2000， 2001–2011	√	√		[83]
	1980s			√	[84]
	2001–2010		√	√	[85]

Table S10. (continued)

Grassland	Existing estimates of C changes at national scale					Reference
	Period	C_{AGB}[d] (kg C ha^{-1} yr^{-1})	C_{BGB}[e] (kg C ha^{-1} yr^{-1})	C_{TB}[f] (kg C ha^{-1} yr^{-1})	C_{SOCD} (kg C ha^{-1} yr^{-1})	
	1982–1999	√	√		√	[82]
	1980s–1990s	√	√			[88]
	1961–2013			√	√	[101]
	1980s–2010s				√	[102]
	1981–2000			√	√	[103]

Grassland	Site-specific observations of C storage and SOC change					Reference
	Years since grazing exclusion	AGB (kg C ha^{-1})	BGB[g] (kg C ha^{-1})	SOCD[h] (kg C ha^{-1})	RC_{SOCD}[i] (% yr^{-1})	
	0, 3, 6, 9, 11	√	√			[89]
	0, 8, 11, 14, 21, 25	√	√			[90]
	0, 26, 31		√			[91]
	0, 2		√			[92]
	0, 2, 7, 13		√			[93]
	0, 5, 10	√	√			[94]
	0, 20, 25, 30	√	√			[95]
	0, 4, 8		√			[96]
	0, 4, 8		√			[97]
	0, 5		√			[98]
	0, 8, 12, 18, 25, 33	√	√			[99]
	1, 2, 3, 4, 5, 6, 7, 8, 9, 10,12, 15, 16, 20, 24, 26				√	[104]
	3, 5			√		[105]
	5			√		[106]

Table S10. (continued)

Grassland	Site-specific observations of C storage and SOC change					Reference
	Years since grazing exclusion	AGB ($kg\ C\ ha^{-1}$)	BGB ($kg\ C\ ha^{-1}$)	SOCD ($kg\ C\ ha^{-1}$)	RC_{SOCD} ($\%\ yr^{-1}$)	
	5, 7, 8, 10, 11, 13, 14, 18, 20, 21, 22, 24, 25			√		[107]
	2, 4, 6, 8, 10			√		[108]

Wetland	Site-specific observations of SOC storage				Reference
	CK and years since restoration	SOC [j] ($g\ kg^{-1}$)	BD [k] ($g\ cm^{-3}$)	SOCD ($kg\ C\ ha^{-1}$)	
	CK, 2	√			[117]
	CK, 1, 3, 5	√			[118]
	CK, 3, 5, 7	√			[119]
	CK, 7, 12, 21			√	[120]
	CK, 3, 15, 30	√			[121]
	CK, 7	√			[122]
	0, 3, 6, 12	√			[123]
	CK, 4, 5, 8, 10	√			[124]
	CK, 7	√			[125]
	0, 2, 15, 30	√	√		[126]
	CK, 8	√	√		[127]
	CK, 4, 14			√	[128]
	CK, 6, 12	√			[129]
	CK, 2, 3, 4, 5, 6	√			[130]
	CK, 5, 10	√			[131]

[a] aboveground biomass; [b] total biomass; [c] changes in soil organic carbon density; [d] changes in aboveground biomass; [e] changes in belowground biomass; [f] changes in total biomass; [g] belowground biomass; [h] soil organic carbon density; [i] relative changes in soil organic carbon density; [j] SOC concentration; [k] soil bulk density. The soil organic carbon density was calculated by SOC concentration and BD [133]. The BD in wetland soils was estimated by a function of SOC concentration [134] when it is not available in the literature.

REFERENCES in SI

1. The People's Republic of China Third National Communication on Climate Change. Beijing, December 2018.
2. Janssens IA, Freibauer A and Ciais P et al. Europe's terrestrial biosphere absorbs 7 to 12% of European anthropogenic CO_2 emissions. *Science* 2003; **300**:1538–1542.
3. Shvidenko A and Nilsson S. A synthesis of the impact of Russian forests on the global carbon budget for 1961-1998. *Tellus Ser B-Chem Phys Meteorol* 2003; **55**: 391–415.
4. Fang JY, Guo ZD and Piao SL et al. Terrestrial vegetation carbon sinks in China, 1981—2000. *Sci China Ser D-Earth Sci* 2007; **50**: 1341–1350.
5. Pan Y, Birdsey RA and Fang J et al. A large and persistent carbon sink in the world's forests. *Science* 2011; **333**: 988–993.
6. Wenzel S, Cox PM and Eyring V et al. Projected land photosynthesis constrained by changes in the seasonal cycle of atmospheric CO_2. *Nature* 2016; **538**: 499–502.
7. Ueyama M, Ichii K, Kobayashi H et al. Inferring CO_2 fertilization effect based on global monitoring land-atmosphere exchange with a theoretical model. *Environ Res Lett* 2020; **15**, 084009 https://doi.org/10.1088/1748-9326/ab79e5.
8. Norby RJ, DeLuciac EH and Gielend B et al. Forest response to elevated CO_2 is conserved across a broad range of productivity. *Proc Natl Acad Sci USA* 2005; **102**: 18052–18056.
9. State Forestry Administration of China. The Forestry Action Plan to Address Climate Change. Beijing: China Forestry Press, 2010.
10. Xu B, Guo ZD and Piao SL et al. Biomass carbon stocks in China's forests between 2000 and 2050: A prediction based on forest biomass–age relationships. *Sci China Life Sci* 2010; **53**: 776–783.
11. Liu Y. Carbon carrying capacity and carbon sequestration potential of global forests based on the integrated analysis of inventory dataset. *Ph.D. Thesis*. The University of Chinese Academy of Sciences, 2013 (in Chinese with English abstract).
12. Qiu Z, Feng Z and Song Y et al. Carbon sequestration potential of forest vegetation in China from 2003 to 2050: Predicting forest vegetation growth based on climate and the environment. *J Clean Prod* 2020; **252**, 119715, https://doi.org/10.1016/j.jclepro.2019.119715.
13. Fang J, Chen A and Peng C et al. Changes in forest biomass carbon storage in China between 1949 and 1998. *Science* 2001; **292**: 2320–2322.
14. Fang J, Guo Z and Piao S et al. Terrestrial vegetation carbon sinks in China, 1981–2000. *Sci China Ser D-Earth Sci* 2007; **50**: 1341–1350.
15. Guo ZD, Hu HF and Li P et al. Spatio-temporal changes in biomass carbon sinks in China's forests from 1977 to 2008. *Sci China Life Sci* 2013; **56**: 661–671.
16. Liu GH, Fu BJ and Fang JY. Carbon dynamics of Chinese forests and its contribution to global carbon balance. *Acta Ecol Sin* 2000; **20**: 733–740 (in Chinese with English abstract).
17. Pan Y, Luo T and Birdsey R et al. New estimates of carbon storage and sequestration in China's forest: effects of age-class and method on inventory-based estimation. *Clim Chang* 2004; **67**: 211–236.
18. Zhao M and Zhou GS. Carbon storage of forest vegetation and its relationship with climatic factors. *Sci Geogr Sin* 2004; **24**: 50–54 (in Chinese with English abstract).
19. Piao SL, Fang JY and Zhu B et al. Forest biomass carbon stocks in China over the past 2 decades: estimation based on integrated inventory and satellite data. *J Geophys Res* 2005; **110**: G01006. https://doi.org/10.1029/2005JG000014.
20. Xu XL, Cao MK and Li KR. Temporal- spatial dynamics of carbon storage of forest vegetation in China.

Prog Geogr 2007; **26**: 1–10 (in Chinese with English abstract).
21. Wu QB, Wang XK and Duan XN et al. Carbon sequestration and its potential by forest ecosystems in China. *Acta Ecol Sin* 2008; **28**: 517–525 (in Chinese with English abstract).
22. Zhang C, Ju W and Chen J et al. China's forest biomass carbon sink based on seven inventories from 1973 to 2008. *Clim Chang* 2013; **118**: 933–948.
23. Zhao MW, Yue TX and Zhao N et al. Spatial distribution of forest vegetation carbon stock in China based on HASM. *Acta Geograph Sin* 2013; **68**: 1212–1224 (in Chinese with English abstract).
24. Tang X, Zhao X and Bai Y et al. Carbon pools in China's terrestrial ecosystems: New estimates based on an intensive field survey. *Proc Natl Acad Sci USA* 2018; **115**: 4021–4026.
25. Sun Z, Peng S and Li X et al. Changes in forest biomass over China during the 2000s and implications for management. *For Ecol Manage* 2015; **357**: 76–83.
26. Zhang YX and Wang X J. Study on forest volume-to-biomass modeling and carbon storage dynamics in China. *Sci Sin Vitae* 2021, **51**: 199–214 (in Chinese with English abstract).
27. Yang Y, Li P and Ding J et al. Increased topsoil carbon stock across China's forests. *Glob Change Biol* 2014; **20**: 2687–2696.
28. Abaker WE, Berninger F and Saiz G et al. Contribution of *Acacia senegal* to biomass and soil carbon in plantations of varying age in Sudan. *For Ecol Manage* 2016; **368**: 71–80.
29. Arevalo CBM, Bhatti JS and Chang SX et al. Ecosystem carbon stocks and distribution under different land-uses in north central Alberta, Canada. *For Ecol Manage* 2009; **257**: 1776–1785.
30. Armolaitis K, Aleinikovienė J and Lubytė J et al. Stability of soil organic carbon in agro and forest ecosystems on Arenosol. *Zemdirbyste* 2013; **100**: 227–234.
31. Bruckman VJ, Yan S and Hochbichler E et al. Carbon pools and temporal dynamics along a rotation period in *Quercus* dominated high forest and coppice with standards stands. *For Ecol Manage* 2011; **262**: 1853–1862.
32. Chen L, Gong J and Fu B et al. Effect of land use conversion on soil organic carbon sequestration in the loess hilly area, Loess Plateau of China. *Ecol Res* 2007; **22**: 641–648.
33. Chen LF, He ZB and Zhu X et al. Impacts of afforestation on plant diversity, soil properties, and soil organic carbon storage in a semi-arid grassland of northwestern China. *Catena* 2016; **147**: 300–307.
34. Cook RL, Binkley D and Mendes JCT et al. Soil carbon stocks and forest biomass following conversion of pasture to broadleaf and conifer plantations in southeastern Brazil. *For Ecol Manage* 2014; **324**: 37–45.
35. DeGryze S, Six J and Paustian K et al. Soil organic carbon pool changes following land-use conversions. *Glob Change Biol* 2004; **10**: 1120–1132.
36. Epron D, Marsden C and M'Bou AT et al. Soil carbon dynamics following afforestation of a tropical savannah with *Eucalyptus* in Congo. *Plant Soil* 2009; **323**: 309–322.
37. Fang HJ, Yu GR and Cheng SL et al. ^{13}C abundance, water-soluble and microbial biomass carbon as potential indicators of soil organic carbon dynamics in subtropical forests at different successional stages and subject to different nitrogen loads. *Plant Soil* 2009; **320**: 243–254.
38. Fataei E and Varamesh S. Carbon stocks in a 20-year-old coniferous plantation-a case study in Fandoghloo region, northwestern Iran. *Appl Ecol Environ Res* 2016;**14**: 325–337.
39. Garay M , Amiotti N and Zalba P. Response of phosphorus pools in Mollisols to afforestation with *Pinus radiata* D. Don in the Argentinean Pampa. *For Ecol Manage* 2018; **422**: 33–40.
40. Gill HS and Abrol IP. Evaluation of coastal sandy soils and their saline ground waters for afforestation: A case study from India. *New For* 1990; **4**: 37–53.

41. Goel V and Behl HM. Selection of *Leucaena* species for afforestation and amelioration of sodic soils. *Land Degrad Dev* 2002; **13**: 387–393.
42. Groenendijk FM, Condron LM and Rijkse WC. Effects of afforestation on organic carbon, nitrogen and sulfur concentrations in New Zealand hill country soils. *Geoderma* 2002; **108**: 91–100.
43. Guo Y, Qin F and Yao Y et al. Effects of land use changes on soil organic carbon and soil microbial biomass carbon in low hills of north Yanshan Mountains. *Range Manag Agrofor* 2014; **35**: 15–21.
44. Hiltbrunner D, Zimmermann S and Hagedorn F. Afforestation with Norway spruce on a subalpine pasture alters carbon dynamics but only moderately affects soil carbon storage. *Biogeochemistry* 2013; **115**: 251–266.
45. Jain RK and Singh B. Biomass production and soil amelioration in a high density *Terminalia Arjuna* plantation on sodic soils. *Biomass Bioenerg* 1998; **15**: 187–192.
46. Jiménez JJ, Lal R and Leblanc HA et al. Soil organic carbon pool under native tree plantations in the Caribbean lowlands of Costa Rica. *For Ecol Manage* 2007; **24**: 134–144.
47. Justine MF, Yang W and Wu F et al. Biomass stock and carbon sequestration in a chronosequence of *Pinus massoniana* plantations in the upper reaches of the Yangtze River. *Forests* 2015; **6**: 3665–3682;
48. Lemma B, Kleja DB and Nilsson I et al. Soil carbon sequestration under different exotic tree species in the southwestern highlands of Ethiopia. *Geoderma* 2006; **136**: 886–898.
49. Li S, Su J and Liu W et al. Changes in biomass carbon and soil organic carbon stocks following the conversion from a secondary coniferous forest to a pine plantation. *PLoS One* 2015; **10**: e0135946. https://doi.org/10.1371/journal.pone.0135946.
50. Makumba W, Akinnifesi FK and Janssen B et al. Long-term impact of a gliricidia-maize intercropping system on carbon sequestration in southern Malawi. *Agric Ecosyst Environ* 2007; **118**: 237–243.
51. Mao R, Zeng DH and Hu YL et al. Soil organic carbon and nitrogen stocks in an age-sequence of poplar stands planted on marginal agricultural land in northeast China. *Plant Soil* 2010; **332**: 277–287.
52. Markewitz D, Sartori F and Craft C. Soil change and carbon storage in longleaf pine stands planted on marginal agricultural lands. *Ecol Appl* 2002; **12**: 1276–1285.
53. McGrath DA, Duryea ML and Cropper WP et al. Soil phosphorus availability and fine root proliferation in Amazonian agroforests 6 years following forest conversion. *Agric Ecosyst Environ* 2001; **83**: 271–284.
54. Mishra A, Sharma SD and Gupta MK. Soil rehabilitation through afforestation: evaluation of the performance of *Prosopis juliflora*, *Dalbergia sissoo* and *Eucalyptus tereticornis* plantations in a sodic environment. *Arid Land Res Manag* 2003; **17**: 257–269.
55. Montagnini F. Accumulation in above-ground biomass and soil storage of mineral nutrients in pure and mixed plantations in a humid tropical lowland. *For Ecol Manage* 2000; **134**: 257–270.
56. Morris SJ, Bohm S and Haile-Mariam S et al. Evaluation of carbon accrual in afforested agricultural soils. *Glob Change Biol* 2007; **13**: 1145–1156.
57. Navarrete IA and Tsutsuki K. Land-use impact on soil carbon, nitrogen, neutral sugar composition and related chemical properties in a degraded ultisol in Leyte, Philippines. *Soil Sci Plant Nutr* 2008; **54**: 321–331.
58. Noble AD, Little IP and Randall PJ. The influence of *Pinus radiata*, *Quercus suber*, and improved pasture on soil chemical properties. *Aust J Soil Res* 1999; **37**: 509–526.
59. Nørnberg P, Sloth L and Nielsen KE. Rapid changes of sandy soils caused by vegetation changes. *Can J Soil Sci* 1993; **73**: 459–468.
60. Ovington JD. Studies of the development of woodland conditions under different trees: IV. the ignition

loss, water, carbon and nitrogen content of the mineral soil. *J Ecol* 1956; **44**: 171–179.

61. Pibumrung P, Gajaseni N and Popan A. Profiles of carbon stocks in forest, reforestation and agricultural land, northern Thailand. *J For Res* 2008; **19**: 11–18.
62. Quideau SA and Bockheim JG. Vegetation and cropping effects on pedogenic processes in a sandy prairie soil. *Soil Sci Soc Am J* 1996; **60**: 536–545.
63. Quinkenstein A, Böhm C and da Silva Matos E et al. Assessing the carbon sequestration in short rotation coppices of *Robinia pseudoacacia* L. on marginal sites in northeast Germany. In: Kumar BM and Nair PKR (eds.). *Carbon Sequestration Potential of Agroforestry Systems: Opportunities and Challenges, Advances in Agroforestry*. Springer Science Business Media B.V., 2011; **8**: 201–216.
64. Resh SC, Binkley D and Parrotta JA. Greater soil carbon sequestration under nitrogen-fixing trees compared with *Eucalyptus* species. *Ecosystems* 2002; **5**: 217–231.
65. Sartori F, Rattan L and Michael HE et al. Changes in soil carbon and nutrient pools along a chronosequence of poplar plantations in the Columbia Plateau, Oregon, USA. *Agric Ecosyst Environ* 2007; **122**: 325–339.
66. Singh B, Tripathi KP and Jain RK et al. Fine root biomass and tree species effects on potential N mineralization in afforested sodic soils. *Plant Soil* 2000;**219**: 81–89.
67. Singh G and Rathod TR. Tree and crop growth and soil resource availability in *Hardwickia binata* Roxb. agroforestry systems in the Indian desert. *Arid Land Res Manag* 2007; **21**: 193–210.
68. Singh YP, Singh G and Sharma DK. Ameliorative effect of multipurpose tree species grown on sodic soils of Indo-Gangetic alluvial plains of India. *Arid Land Res Manag* 2011; **25**: 55–74.
69. Swamy SL and Puri S. Biomass production and C-sequestration of *Gmelina arborea* in plantation and agroforestry system in India. *Agrofor Syst* 2005; **64**: 181–195.
70. Tabari M and Salehi A. Soil carbon sequestration potential of eldar pine and black locust afforestation in a semi-arid zone of Iran. *Res J Environ Sci* 2008; **2**: 483–490.
71. Ulery AL, Graham RC and Chadwick OA et al. Decade-scale changes of soil carbon, nitrogen and exchangeable cations under chaparral and pine. *Geoderma* 1995; **65**: 121–134.
72. Ussiri DAN, Lal R and Jacinthe PA. Soil properties and carbon sequestration of afforested pastures in reclaimed minesoils of Ohio. *Soil Sci Soc Am J* 2006; **70**: 1797–1806.
73. Wang K, Deng L and Ren Z et al. Dynamics of ecosystem carbon stocks during vegetation restoration on the Loess Plateau of China. *J Arid Land* 2016; **8**: 207–220.
74. Wang T, Kang FF and Cheng XQ et al. Soil organic carbon and total nitrogen stocks under different land uses in a hilly ecological restoration area of north China. *Soil Tillage Res* 2016; **163**: 176–184.
75. Wei X, Qiu L and Shao M et al. The accumulation of organic carbon in mineral soils by afforestation of abandoned farmland. *PLoS One* 2012; **7**: e32054. https://doi:10.1371/journal.pone.0032054
76. Wei XH, Li QL and Liu YQ et al. Restoring ecosystem carbon sequestration through afforestation: a sub-tropic restoration case study. *For Ecol Manage* 2013; **300**: 60–67.
77. Xie J, Guo J and Yang Z et al. Rapid accumulation of carbon on severely eroded red soils through afforestation in subtropical China. *For Ecol Manage* 2013; **300**: 53–59.
78. Yan M, Zhang X and Zhou G et al. Temporal and spatial variation in soil respiration of poplar plantations at different developmental stages in Xinjiang, China. *J Arid Environ* 2011; **75**: 51–57.
79. Zhang K, Dang H and Tan S et al. Vegetation community and soil characteristics of abandoned agricultural land and pine plantation in the Qinling Mountains, China. *For Ecol Manage* 2010; **259**: 2036–2047.
80. Zhiyanski M, Glushkova M and Ferezliev A et al. Carbon storage and soil property changes following

afforestation in mountain ecosystems of the Western Rhodopes, Bulgaria. *iForest* 2016; **9**: 626–634.

81. Zinn YL, Resck DVS and da Silva JE. Soil organic carbon as affected by afforestation with *Eucalyptus* and *Pinus* in the Cerrado region of Brazil. *For Ecol Manage* 2002; **166**: 285–294.
82. Piao S, Fang J and Ciais P et al. The carbon balance of terrestrial ecosystems in China. *Nature* 2009; **458**:1009–1014.
83. Xie ZX, Tang ZY and Liu Q et al. *Study on the Carbon Budget in China's Shrubland Ecosystem*. Beijing: Science Press, 2020 (in Chinese).
84. Wang S, Tian H and Liu J et al. Pattern and change of soil organic carbon storage in China: 1960s–1980s. *Tellus B* 2003; **55**: 416–427.
85. Fang J, Yu G and Liu L et al. Climate change, human impacts, and carbon sequestration in China. *Proc Natl Acad Sci USA* 2018; **115**: 4015–4020.
86. Bai Y, Zhao Y and Wang Y et al. Assessment of ecosystem services and ecological regionalization of grasslands support establishment of ecological security barriers in Northern China. *Bull Chin Acad Sci* 2020; **35**: 675–689 (in Chinese with English abstract).
87. Hu Z, Li S and Guo Q et al. A synthesis of the effect of grazing exclusion on carbon dynamics in grasslands in China. *Glob Change Biol* 2016; **22**: 1385–1393.
88. Piao S, Fang J, Zhou L et al. Changes in biomass carbon stocks in China's grasslands between n1982 and 1999. *Global Biogeochem Cycles* 2007; **21**, GB2002, doi:10.1029/2005GB002634.
89. Li W, Liu Y and Wang J et al. Six years of grazing exclusion is the optimum duration in the alpine meadow-steppe of the northeastern Qinghai-Tibetan Plateau. *Sci Rep* 2018; **8**: 17269 DOI:10.1038/s41598-018-35273-y.
90. Ao Y, Jiao Y and Xu Z. The changes of carbon and nitrogen storage of plant-soil system of enclosure years in typical steppe. *Ecol Environ Sci* 2011; **20**(10): 1403–1410 (in Chinese with English abstract).
91. Zhai XJ, Huang D and Wang K. Effects of fencing and grazing on vegetation and soil in typical grassland. *Chinese Journal of Grassland* 2015; **37**(6): 73–78 (in Chinese with English abstract).
92. Dong XY, Fu H and Li XD et al. Effects on plant biomass and CNP contents of plants in grazed and fenced steppe grasslands of the Loess Plateau. *Acta Prataculturae Sinica* 2010; **19**(2): 175–192 (in Chinese with English abstract).
93. Fan YG, Hu YK and Li KH et al. Effects of different disturbances on the diversity and biomass of the phytobiocoenoses in alpine steppes. *Arid Zone Research* 2008; **25**(4): 531–536 (in Chinese with English abstract).
94. Fan YJ, Hou XY and Shi HX et al. The response of carbon reserves of plants and soils to different grassland managements on alpine meadow of three headwater source regions. *Grassland and Turf* 2012; **32**(5): 41–46 (in Chinese with English abstract).
95. He NP, Han XG and Yu GR. Carbon and nitrogen sequestration rate in long-term fenced grasslands in Inner Mongolia, China. *Acta Ecologica Sinica* 2011; **31**(15): 4270–4276 (in Chinese with English abstract).
96. Hong JD, Wu JB and Wang XD. Effects of grazing and fencing on *Stipa purpurea* community biomass allocation and carbon, nitrogen, and phosphorus pools on the northern Tibet Plateau alpine. *Pratacultural Science* 2015; **32**(11): 1878–1886 (in Chinese with English abstract).
97. Wu JB and Wang XD. Effects of enclosure ages on community characters and biomass of the degraded alpine steppe at the northern Tibet. *Acta Agrestia Sinica* 2017; **25**(2): 261–266 (in Chinese with English abstract).
98. Xijile, Zhao ML and Wang J et al. Effect of enclosure on carbon storage in rangeland. *Journal of Inner*

Mongolia Forestry Science & Technology 2012; **38**(3): 8–12 (in Chinese with English abstract).

99. Jing G. Responses of grassland community structure and functions to management practices on the semi-arid area of Loess Plateau. *Ph.D. Thesis*. The University of Chinese Academy of Sciences, 2017 (in Chinese with English abstract).
100. Liu X, Wang Z and Zheng K et al. Changes in soil carbon and nitrogen stocks following degradation of alpine grasslands on the Qinghai-Tibetan Plateau: A meta-analysis. *Land Degrad Dev* 2021; **32**: 1262–1273.
101. Zhang L, Zhou GS and Ji YH et al. Spatiotemporal dynamic simulation of grassland carbon storage in China. *Sci China Ser D-Earth Sci* 2016; **59**(10): 1946–1958.
102. Xu L, Yu G and He N. Increased soil organic carbon storage in Chinese terrestrial ecosystems from the 1980s to the 2010s. *J Geogr Sci* 2019; **29**(1): 49–66.
103. Tian H, Melillo J and Lu C et al. China's terrestrial carbon balance: Contributions from multiple global change factors. *Global Biogeochem Cycles* 2011; **25**, GB1007, doi:10.1029/2010GB003838.
104. Xiong D, Shi P and Zhang X et al. Effects of grazing exclusion on carbon sequestration and plant diversity in grasslands of China—A meta-analysis. *Ecol Eng* 2016; **94**: 647–655.
105. Zhu G, Guo N and Lü G et al. Effects of enclosure on soil physiochemical properties and stable carbon and nitrogen isotopes in Inner Mongolia desert steppe. *Soils* 2020; **52**(4): 840–845 (in Chinese with English abstract).
106. Yang Y, Liu AJ and Li LH et al. Effects of fencing on vegetation community characteristics and soil properties of a typical steppe in Inner Mongolia. *Acta Prataculturae Sinica* 2016; **25**(5): 21–29 (in Chinese with English abstract).
107. Ao Y. Study on soil ecological stoichiometry of enclosing life in typical steppe. *M.S. Thesis*. Inner Mongolia Normal University, 2012 (in Chinese with English abstract).
108. Zhang P. Studies on soil organic carbon sequestration in grassland of north China. *Postdoctoral Report*. Institute of Atmospheric Physics, Chinese Academy of Sciences, 2013(in Chinese with English abstract).
109. Pacala SW, Hurtt GC and Baker D et al. Consistent land- and atmosphere-based US carbon sink estimates. *Science* 2001; **292**: 2316–2320.
110. Huang Y, Yu YQ and Zhang W et al. Agro-C: A biogeophysical model for simulating the carbon budget of agroecosystems. *Agric For Meteorol* 2009; **149**(1): 106–129.
111. Yu YQ, Huang Y and Zhang W. Modeling soil organic carbon change in croplands of China, 1980–2009. *Glob Planet Change* 2012; **82–83**: 115–128.
112. Shi XZ, Yang RW and Weindorf DC et al. Simulation of organic carbon dynamics at regional scale for paddy soils in China. *Clim Change* 2010; **102**: 579–593.
113. Wang G, Huang Y and Wang E et al. Modeling soil organic carbon change across Australian wheat growing areas, 1960-2010. *PLoS One* 2013, **8**(5): e63324. doi:10.1371/journal.pone.0063324
114. National Forestry and Grassland Administration of China. The 13th Five-Year Plan for Wetland Protection. Beijing, November 2016.
115. National Forestry and Grassland Administration of China. National Planning for Wetland Protection (2002-2030). Beijing, September 2003.
116. Villa JA and Bernal B. Carbon sequestration in wetlands, from science to practice: An overview of the biogeochemical process, measurement methods, and policy framework. *Ecol Eng* 2018; **114**: 115–128.
117. Yang Q, Tian K and Xiao D et al. Ecological restoration effect of closed and half-closed degraded wetlands in Northwest Yunnan Plateau, Southwest China. *Chin J Appl Ecol* 2012; **23**(6): 1520–1526 (in Chinese with English abstract).

118. Wu Z, Jin H and Wang F. Eco-restoration of degenerative wetland in AIBI lake region. *J Soil Water Conserv* 2012; **26**:211–221 (in Chinese with English abstract).
119. Dong KK, Wang H and Yang LY et al. Change characteristics of soil carbon and nitrogen contents in the Yellow River Delta soil after artificial restoration. *Acta Ecol Sin* 2011; **31**(16): 4778–4782 (in Chinese with English abstract) .
120. Yang YL, Mou XJ and Wen BL et al. Soil carbon, nitrogen and phosphorus concentrations and stoichiometries across a chronosequence of restored inland soda saline-alkali wetlands, western Songnen Plain, Northeast China. *Chin Geogra Sci* 2020; **30**(5): 934–946.
121. Yang L, Jiang M and Zhu WH et al. Soil bacterial communities with an indicative function response to nutrients in wetlands of Northeastern China that have undergone natural restoration. *Ecol Indic* 2019; **101**: 562–571.
122. Wang H, Wang RQ and Yu Y et al. Soil organic carbon of degraded wetlands treated with freshwater in the Yellow River Delta, China. *J Environ Manage* 2011; **92**: 2628–2633.
123. Song YY, Song CC and Yang GS et al. Changes in labile organic carbon fractions and soil enzyme activities after marshland reclamation and restoration in the Sanjiang Plain in northeast China. *Environ Manage* 2012; **50**(3): 418–426.
124. Ren H, Jian SG and Lu HF et al. Restoration of mangrove plantations and colonisation by native species in Leizhou bay, South China. *Ecol Res* 2008; **23**(2): 401–407.
125. Liu XY, Tao KY and Sun J et al. The introduction of woody plants for freshwater wetland restoration alters the archaeal community structure in soil. *Land Degrad Develop* 2017; **28**(7): 1933–1942.
126. Guo J, Jiang HB and Bian HF et al. Natural succession is a feasible approach for cultivated peatland restoration in Northeast China. *Ecol Eng* 2017; **104**: 39–44.
127. Gao JQ, Zhang XW and Lei GC et al. Soil organic carbon and its fractions in relation to degradation and restoration of wetlands on the Zoige Plateau, China. *Wetlands* 2014; **34**(2): 235–241.
128. Feng JX, Zhou J and Wang LM et al. Effects of short-term invasion of *Spartina alterniflora* and the subsequent restoration of native mangroves on the soil organic carbon, nitrogen and phosphorus stock. *Chemosphere* 2017; **184**: 774–783.
129. Ding XL, Zhang B and Filley TR et al. Changes of microbial residues after wetland cultivation and restoration. *Biol Fert Soils* 2019; **55**(4): 405–409.
130. Cui BS, Yang QC and Yang ZF et al. Evaluating the ecological performance of wetland restoration in the Yellow River Delta, China. *Ecol Eng* 2009; **35**(7)**:** 1090–1103.
131. Chen GC and Ye Y. Restoration of *Aegiceras corniculatum* mangroves in Jiulongjiang Estuary changed macro-benthic faunal community. *Ecol Eng* 2011; **37**(2): 224–228.
132. Qin Z, Huang Y and Zhuang Q et al. Soil organic carbon sequestration potential of cropland in China. *Glob Biogeochem Cycles* 2013; **27**:711–722.
133. Sun W, Huang Y and Zhang W et al. Carbon sequestration and its potential in agricultural soils of China. *Glob Biogeochem Cycles* 2010; **24**, GB3001, doi:10.1029/2009GB003484
134. Huang Y, Sun WJ and Zhang W et al. Marshland conversion to cropland in northeast China from 1950 to 2000 reduced the greenhouse effect. *Glob Change Biol* 2010, **16**:680–695.
